# Targeted degradation of EGFR 19Del by PROTACs suppresses tumor growth in non-small-cell lung cancer

**DOI:** 10.3389/fphar.2025.1604661

**Published:** 2025-10-24

**Authors:** Lianhua Piao, Ying Gao, Yangyang Su, Qihui Li, Xiaofeng Yuan, Wangqiu He, Wanzhou Zhao, Janne Kulpakko, Pei-Chieng Cha, Shan Chang, Ren Kong

**Affiliations:** ^1^ Institute of Bioinformatics and Medical Engineering, Jiangsu University of Technology, Changzhou, Jiangsu, China; ^2^ Primary Biotechnology Co., Ltd., Suzhou, China; ^3^ Department of Orthopaedics, The Third Affiliated Hospital of Soochow University, Changzhou, Jiangsu, China; ^4^ The Nanjing Han & Zaenker Cancer Institute (NHZCI), OG Pharmaceuticals, Nanjing, Jiangsu, China; ^5^ Aqsens Health Oy, Turku, Finland; ^6^ Department of Genomic Medicine, Research Institute, National Cerebral and Cardiovascular Center, Suita, Japan

**Keywords:** EGFR^De19^, epidermal growth factor receptor, EGFR^L858R^, non-small-cell lung cancer, proteolysis-targeting chimeras, molecular docking

## Abstract

**Object:**

The occurrence of acquired resistance to epidermal growth factor receptor (EGFR) tyrosine kinase inhibitors (TKIs) poses a significant challenge in treating non-small-cell lung cancer (NSCLC), limiting the clinical use of EGFR-TKIs. Proteolysis-targeting chimeras (PROTACs) demonstrate promise in preclinical settings. This study is aimed to design effective EGFR degraders by linking CRBN ligands with relatively lower molecular weights.

**Methods:**

Computational methods are employed to do the rational design for the PROTACs. Western blots are used to examine the expression of proteins including EGFR. Cell viability and colony-formation assays are conducted to evaluate the anti-proliferative effects of degraders to NSCLC cell lines, and apoptosis assays are assessed by Annexin V‐FITC/PI dual staining followed by flow cytometry. Female BALB/c nude mice bearing HCC827 xenografts are administered compound 14 (30 mg/kg) by intraperitoneal injection and the tumor volume and weights are measured.

**Results:**

We designed and synthesized a series of highly potent degraders based on the first‐generation EGFR‐TKI gefitinib and a cereblon (CRBN) ligand. Among these degraders, compound 14, with a relatively low molecular weight of 814.32 Da, exhibits notable activity against EGFR^Del19^ and EGFR^L858R^, with DC50 values of 0.26 nM and 20.57 nM, respectively, while showing no effect on EGFRwt. Additionally, downstream signaling pathways are significantly inhibited. Mechanistic studies indicate that EGFR degradation depends on the ubiquitin–proteasome system (UPS). Furthermore, compound 14 markedly suppresses the growth of HCC827 cells and induces apoptosis, with a 96‐h IC50 value of 4.91 nM, while not affecting the viability of H1299, HeLa, and H1975 cells up to 1 μM. In the HCC827 cell-derived xenograft model, compound 14 demonstrates substantial anti-tumor activity and effectively reduces EGFR^Del19^ protein levels in vivo.

**Conclusion:**

With its low molecular weight and excellent in vitro and in vivo efficacy, compound 14 could serve as a promising lead for developing degrader-based therapies targeting mutated EGFR.

## Introduction

The epidermal growth factor receptor (EGFR, also known as HER1 and ERBB1) is a transmembrane protein receptor that constitutes one of the four members of the ErbB family. Activation of EGFR leads to the initiation of a cascade of downstream signaling pathways and directly regulates cell proliferation and survival. Activating mutations in the EGFR gene are present in approximately 10%–35% of non-small-cell lung cancer (NSCLC) patients, and in-frame deletion in exon 19 (LREA deletions) and substitutions in exon 21 (L858R) account for more than 85% of known EGFR alterations (Westover et al., 2018; Kobayashi and Mitsudomi, 2016). EGFR tyrosine kinase inhibitors (TKIs), such as first-generation gefitinib and erlotinib, second-generation afatinib, and third-generation osimertinib, provide significant clinical benefit in patients with EGFR-mutated NSCLC. However, the occurrence of acquired resistance limits the long-term efficacy of these TKIs. The acquired T790M mutation in exon 20 upon treatment with first- and second-generation TKIs leads to more than 60% resistance. Osimertinib, the third-generation EGFR TKI, irreversibly inhibits mutated EGFR alleles, including T790M, and presents impressive outcomes in EGFR-mutated NSCLC (Soria et al., 2018). However, numerous secondary EGFR mutations, such as C797, G796, L792, L718, and G724 site mutations, have evolved to evade inhibition and mediate resistance to osimertinib in NSCLC harboring EGFR T790M mutants (Thress et al., 2015; Zheng et al., 2017; Fuchs et al., 2021; Chen et al., 2017; Pisapia et al., 2019; Bersanelli et al., 2016; Peled et al., 2017). Hence, novel technologies and approaches to overcome therapeutic resistance to EGFR-TKIs in NSCLC are a priority.

Recently, proteolysis-targeting chimeras (PROTACs) have emerged as a promising modality for targeted protein degradation (TPD) via the ubiquitin–proteasome system (UPS). PROTACs are hetero-bifunctional molecules that are composed of a ligand for targeting the protein of interest (POI), an E3 ubiquitin ligase ligand, and a linker connecting these two ligands (Bekes et al., 2022). Different from traditional inhibitors based on occupancy-driven pharmacology, PROTACs induce protein degradation through an event-driven mechanism and are considered capable of therapeutically targeting a broad range of intractable proteins (Lai and Crews, 2017). Proteins that have evolved resistance mutations to targeted therapies are considered suitable PROTAC targets, and the design of specific PROTAC molecules against mutant EGFR proteins in EGFR mutant-driven NSCLC may be a promising strategy to eradicate the occurrence of secondary mutations.

In the past 10 years, multiple PROTACs targeting EGFRs have been reported. In 2018, a gefitinib-based PROTAC, **compound 3**, which recruits von Hippel–Lindau (VHL) to EGFR, was reported to effectively degrade both exon 19 deletion EGFR and the L858R mutation with DC_50_ (half-maximal degradation concentration) values of 11.7 nM and 22.3 nM, respectively (Burslem et al., 2018). In 2020, **MS39**, a VHL-recruiting EGFR degrader, and **MS154**, a cereblon (CRBN)-recruiting degrader, were discovered to degrade EGFR^Del19^ (DC_50_ = 5.0 nM and 11 nM, respectively) and the L858R point mutant EGFR (DC_50_ = 3.3 nM and 25 nM, respectively) (Cheng et al., 2020). Despite good degradation activity, these two gefitinib-based PROTACs have not displayed impressive IC_50_ values for cell proliferation, probably due to the poor cell permeability. Using the fourth-generation EGFR TKIs as the ligand, Zhang et al. (2020) discovered a CRBN-based **PROTAC 2** and a VHL-based **PROTAC 10**, which have degraded EGFR^Del19^ with DC_50_ values of 45.2 nM and 34.8 nM, respectively, and showed selective cytotoxic activity in HCC827 cells harboring an exon 19 deletion EGFR mutant with IC_50_ values of 180 nM and 220 nM, respectively. Thereafter, by replacing the EGFR ligand part as a reversible EGFR TKI with a purine scaffold, they have reported a more promising degrader **P3** with a DC_50_ value of 0.51 nM and an IC_50_ value of 0.76 nM in HCC827 cells (Zhao et al., 2020). In 2022, they found an osimertinib- and VHL-based covalent EGFR^Del19^ and EGFR^L858R+T790M^ degrader, **CP17**, which exhibited excellent activities against EGFR^Del19^ in HCC827 cells with a DC_50_ value of 0.49 nM and an IC_50_ value of 1.6 nM (Zhao et al., 2022). In 2021, the degrader **SIAIS125**, based on the EGFR irreversible inhibitor canertinib and a CRBN ligand, has also degraded EGFR^Del19^ with a DC_50_ value of 100 nM and an IC_50_ value of 2.6 nM in PC9 cells (Qu et al., 2021). Shi et al. (2022) synthesized a dacomitinib- and VHL-based EGFR^Del19^ degrader, **compound 13**, with a DC_50_ value of 3.57 nM and an IC_50_ value of 6 nM in HCC827 cells. Notably, among these PROTACs, most PROTACs with promising degradation ability and cytotoxic activity have been designed to recruit VHL.

As is well known, one of the disadvantages of PROTACs is their poor cell or tissue permeability due to their large molecular weights (M.W.). Reducing the M.W. to below 1,000 Da can enhance permeability. Therefore, we aim to design effective EGFR degraders by linking CRBN ligands with relatively lower molecular weights. By using computational methods, we designed novel PROTACs based on the first-generation EGFR TKI gefitinib and a CRBN ligand. The most active compound, **14**, has a molecular weight as low as 814.32 Da and potently and selectively degrades EGFR^Del19^ and EGFR^L858R^, but not the wild-type EGFR. This compound-induced degradation occurs in a dose- and time-dependent manner through the ubiquitin–proteasome system. Furthermore, compound **14** significantly inhibits the growth of HCC827 cells harboring the EGFR^Del19^ mutation, both *in vitro* and *in vivo*. The selective and potent EGFR degraders with low molecular weights reported here may serve as lead compounds for treating EGFR mutation-related diseases, such as lung cancer.

## Materials and methods

### Molecular modeling

**FIGURE 1:**
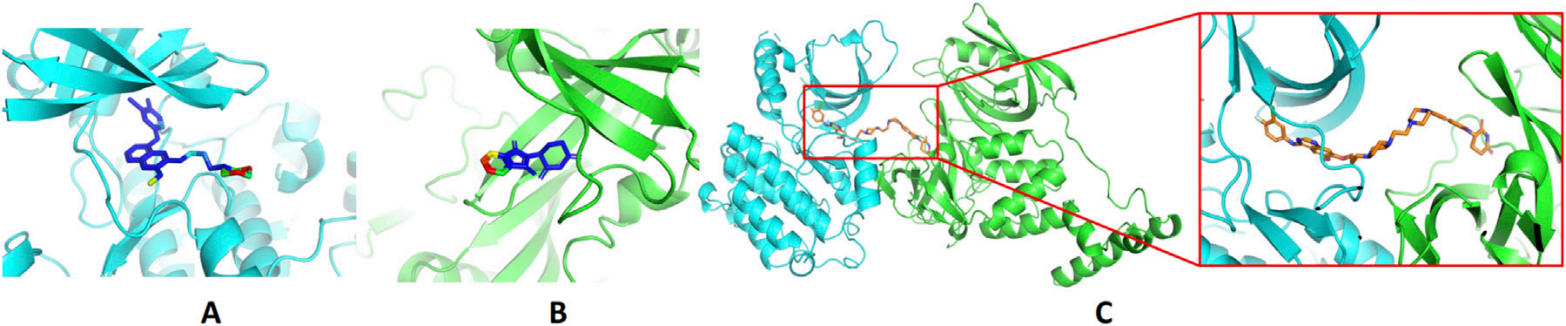
Design of EGFR PROTACs. **(A)** Binding of gefitinib with EGFR (PDB ID: 4I22). **(B)** Binding of thalidomide with CRBN (PDB ID: 6BN7). The proteins are shown in the cartoon model and compounds in the stick model. The compounds are colored by the solvent-accessible area, from blue to red indicating the increase in the solvent-accessible area. **(C)** The predicted ternary structures of EGFR, compound 14, and CRBN.

The PDB files of the EGFR–gefitinib complex structure, 4I22, and the CRBN–thalidomide complex structure, 6BN7, were downloaded from the Protein Data Bank (https://www.rcsb.org/). Water and ions were removed from the structures, and only the proteins and ligands were retained. CoDockPP was used to perform docking between the EGFR–gefitinib complex and the CRBN–thalidomide complex (Kong et al., 2019). Site-constraint search was used with a distance constraint of 20 Å between the tethering atoms from each ligand (Figure 1). A knowledge-based scoring function was used to rank the binding poses. Finally, the top 100 energetically favorable poses were retained.

### Cell lines and cell culture

HCC827 and H1975 cell lines were purchased from ATCC (United States) and cultured according to the manuals. Cells were grown in monolayers in appropriate media supplemented with 10% fetal bovine serum and 1% antibiotic/antimycotic solution. Ba/F3 cells stably expressing EGFR^L858R^ were obtained by infection with lentivirus produced by co-transfection of pLVX-EGFR^L858R^, psPAX2, and pMD2.G into 293T cells, followed by selection with puromycin.

### Cell viability and colony formation assays

Cells (5 × 10^3^ for 48 h or 1 × 10^3^ for 96 h) were seeded in 96-well plates, allowed to attach overnight, and then incubated with various concentrations of compounds for 48 h or 96 h. The viability of cells was determined using Cell Counting Kit-8 (CCK-8; Dojindo), and IC_50_ was calculated using GraphPad Prism (version 6.0). For colony formation assays, cells (1 × 10^3^ cells/well) seeded in 24-well plates were treated with different concentrations of compounds and replaced with fresh media with compounds every 4 days. After 7 days, the cells were washed with PBS, fixed with methanol, and stained with 0.1% crystal violet solution.

### Western blot analysis

Cells at 70%–90% confluence were lysed in RIPA buffer with complete protease inhibitor cocktail (Roche Applied Science, Penzberg, Germany). Lysates were quantified, separated on SDS-PAGE gels, and transferred onto PVDF membranes, followed by the blockade with 5% milk in TBS+ 0.1% Tween-20 buffer for 1 h at room temperature. The following primary antibodies were used: anti-EGFR (1:50000, ABclonal Technology, Wuhan, China), anti-pEGFR (Tyr1068, 1, Cell Signaling Technology, MA, United States), anti-AKT (1:10000, ABclonal Technology, Wuhan, China), anti-pAKT (1:10000, ABclonal Technology, Wuhan, China), and anti-β-actin (1:10000, ABclonal Technology, Wuhan, China). Protein bands were detected using Tanon High-signal ECL Western Blotting Substrate (Tanon Science and Technology Co., Ltd., Shanghai, China). The band density of the protein of interest was quantified using ImageJ.

### Apoptosis assay

HCC827 cells incubated with control vehicle (DMSO), 10 nM of compound 14, or 10 nM of gefitinib for 48 h were collected, stained with FITC Annexin V and propidium iodide (PI), and analyzed using a BD FACSAria II Flow Cytometer. The percentage of apoptotic cells in total cells was designated as the apoptotic index.

### *In vivo* xenograft research

Female BALB/c nude mice (5–6 weeks old) were purchased from Changzhou Cavens Laboratory Animal Co., Ltd. (China) and housed under pathogen-free conditions. HCC827 cells (5 × 10^6^) suspended in 150 μL PBS were inoculated subcutaneously on the left flank of the mouse. When tumor volume reached an average of 70–80 mm^3^, mice were randomly assigned to two groups (three mice in each group). Compound 14 (30 mg/kg, dissolved in 40% PEG 400 and 5% Tween-80) or vehicle (40% PEG 400 and 5% Tween-80) was administered to mice via intraperitoneal injection every 2 days for 21 days. The tumor volume was calculated as follows: tumor volume (mm^3^) = (length × width^2^)/2. All animal experiments were approved by the Ethics Committee for laboratory animals, The Nanjing Han & Zaenker Cancer Institute (File number: OGKQSPF/SQ-112).

### Statistical analysis

All experiments were repeated at least three times independently with similar results, and the results are presented as the mean values ±standard deviation (SD). The significance of differences among groups was assessed using ordinary one-way ANOVA with multiple-comparisons tests using Prism 8 (*p < 0.05, **p < 0.01, ***p < 0.001, and ****p < 0.0001). *p* < 0.05 was considered to indicate a statistically significant difference.

## Results and discussion

### Rational design of EGFR PROTACs

Molecular docking methods were used to predict the ternary complex structure of the POI-PROTAC-E3 ligase in previous studies (Pereira et al., 2023; Ignatov et al., 2023; Zaidman et al., 2020). In this study, computational methods were used for the rational design of PROTACs before conducting costly and time-consuming synthesis experiments. The complex structure of gefitinib with the L858R/T790M mutant EGFR (PDBID: 4I22) was chosen as the POI structure for docking (Gajiwala et al., 2013). By analyzing the solvent availability of the atoms in gefitinib, we selected the solvent-exposed methoxyl group as the attachment point (Figure 1A; Ribeiro et al., 2019). Considering synthesis feasibility, the carbon atom was removed, and the oxygen atom was used as a tethering atom to connect with potential linkers (Figure 1). For the E3 ligase part, the complex structure of thalidomide with CRBN (PDBID: 6BN7) was extracted and used for docking (Nowak et al., 2018). Accordingly, the solvent-available atoms from thalidomide were selected as the tethering atoms (Figure 1). Protein docking was performed using CoDockPP, setting a distance constraint of 20 Å between the tethering atoms from the warhead and the E3 ligand (Kong et al., 2019). The top 100 complex poses, ranked by a knowledge-based scoring function, were retained for further analysis (Supplementary Figure S1). For the top 100 poses, the distance distribution between the tethering atoms is shown in Supplementary Table S1. No pose satisfied with a distance of less than 7 Å, and the largest fraction of poses fell within an atom distance of 15 to 20 Å. We hypothesized that the ideal linker length should be tolerant, allowing as many energetically favorable poses as possible to stabilize the ternary structure. As the C–C bond length was approximately 1.5 Å, we designed and synthesized a series of compounds with different linker length composed of approximately 6–15 atoms, as shown in Table 1. Detailed information on the synthesis and characterization of the compounds is provided in the Supplementary Schemes.

**TABLE 1:** Degradation rates of EGFR under the treatment of compounds 1–15.

ID	2D structure	EGFR degradation (%)^a^
1	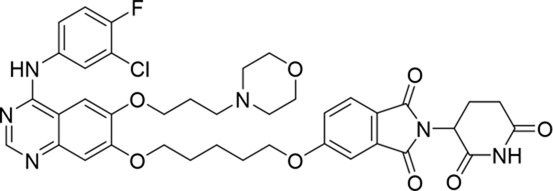	−6.6 ± 4.2
2	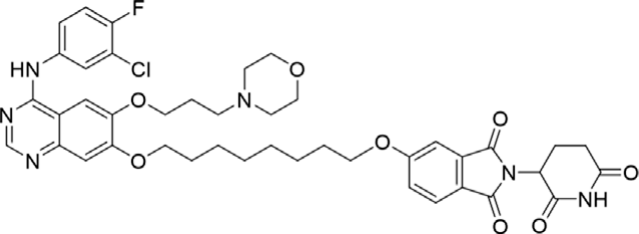	15.3 ± 5.9
3	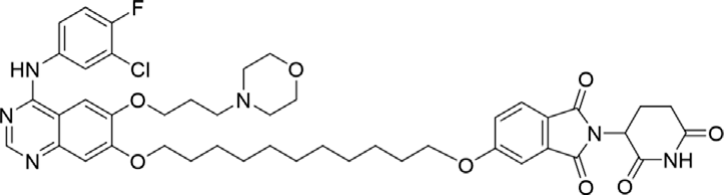	7.0 ± 9.3
4	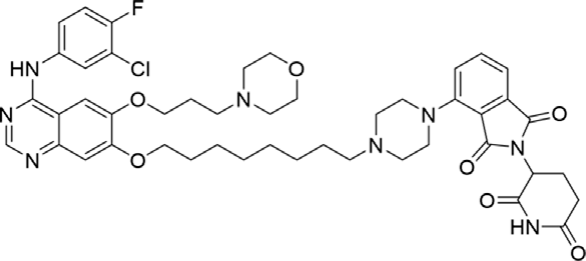	NA
5	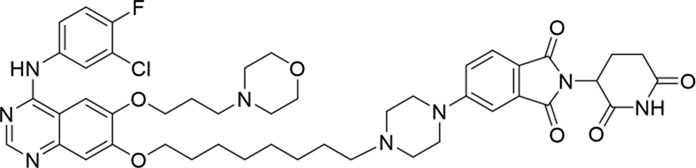	NA
6	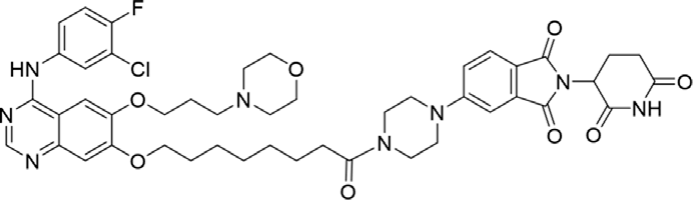	15.0 ± 4.0
7	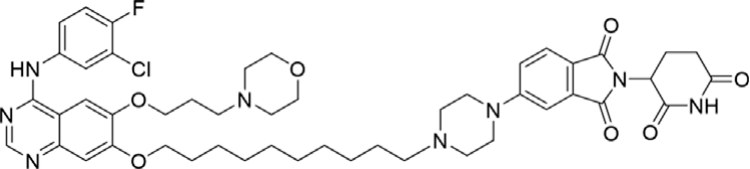	19.4 ± 15.1
8	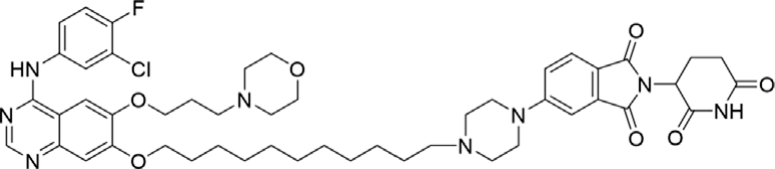	NA
9	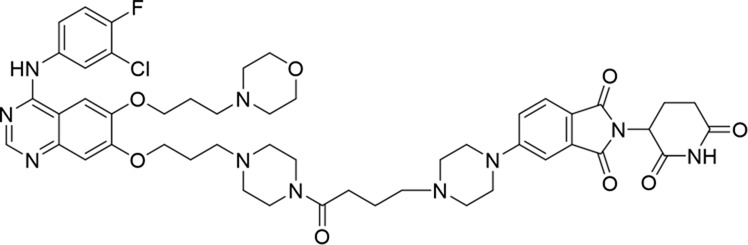	66.3 ± 22.7
10	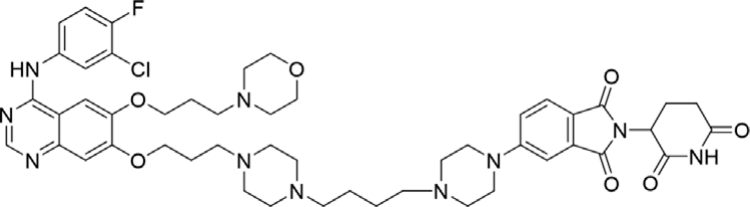	32.4 ± 17.1
11	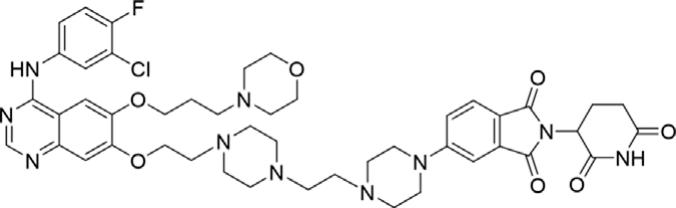	25.1 ± 11.2
12	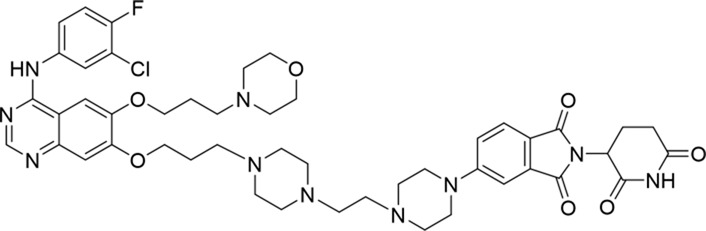	60.0 ± 6.9
13	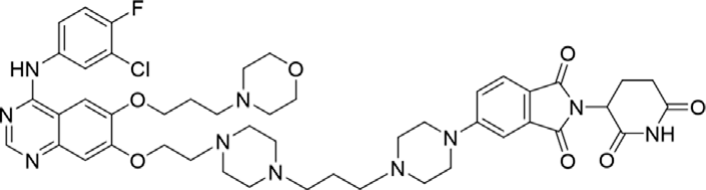	16.8 ± 10.5
14	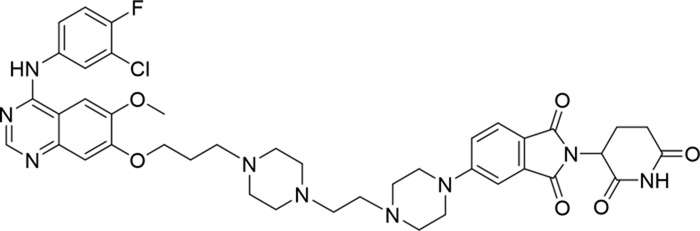	66.3 ± 3.5
15	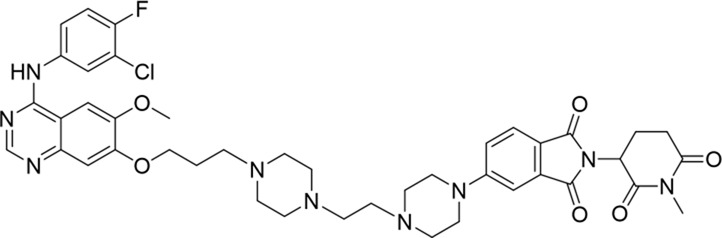	NA

^a^ Average of two independent experiments at 10 nM in HCC827 for 24 h.

NA, not active.

### Initial evaluation of degradation potency of compounds 1–15

The degradation rates of compounds **1–15** at 10 nM were evaluated in HCC827 (EGFR^Del19^) cells, as shown in Table 1. To connect the tethering atoms with different lengths of alkyl chains, we synthesized compounds **1–3**. The short linker with six carbon atoms in compound **1** exhibited no degradation effect, consistent with the distance distribution of the tethering atoms. As shown in Supplementary Table S1, when the distance between the tethering atoms was less than 11 Å, only a limited number of energetically favorable protein–protein poses satisfied this distance condition. With an increased linker length, compounds **2** and **3** showed weak degradation activities. To reduce linker flexibility, piperazine and carbonyl groups were introduced into the compound design. Among these, compounds **6**, **7**, and **9–13**, which featured sufficiently long and relatively rigid linkers, demonstrated significantly enhanced degradation activities.

Based on the binding mode of gefitinib with EGFR (PDBID: 4I22), limited interactions were observed between the morpholine ring and the protein (Gajiwala et al., 2013). Therefore, the extended morpholine ring, which was intended to improve solubility, was removed from compound **12** to reduce molecular weight. The resulting compound **14** exhibited comparable degradation activity to compound **12** (66.3% ± 3.5% for **14** versus 60.0% ± 6.9% for **12**, Table 1) with significantly decreased molecular weight. Compound **15**, which incorporated an extra methyl group on the glutarimide moiety of thalidomide in compound **14,** completely lost its degradation ability (Table 1). It is reported that methylation at this position entirely disrupts the binding of thalidomide to cereblon (Fischer et al., 2014), implying that E3 ligase binding is essential for EGFR degradation. The predicted ternary structure of the most active molecule, compound **14**, is illustrated in Figure 1C. Compound **14** presents an appropriate conformation to bind to the top 1 docking pose of EGFR and CRBN, indicating its potential to induce ternary structure formation.

### Compounds 12 and 14 efficiently decrease EGFR^Del19^ and EGFR^L858R^ proteins but not EGFR^wt^

**FIGURE 2:**
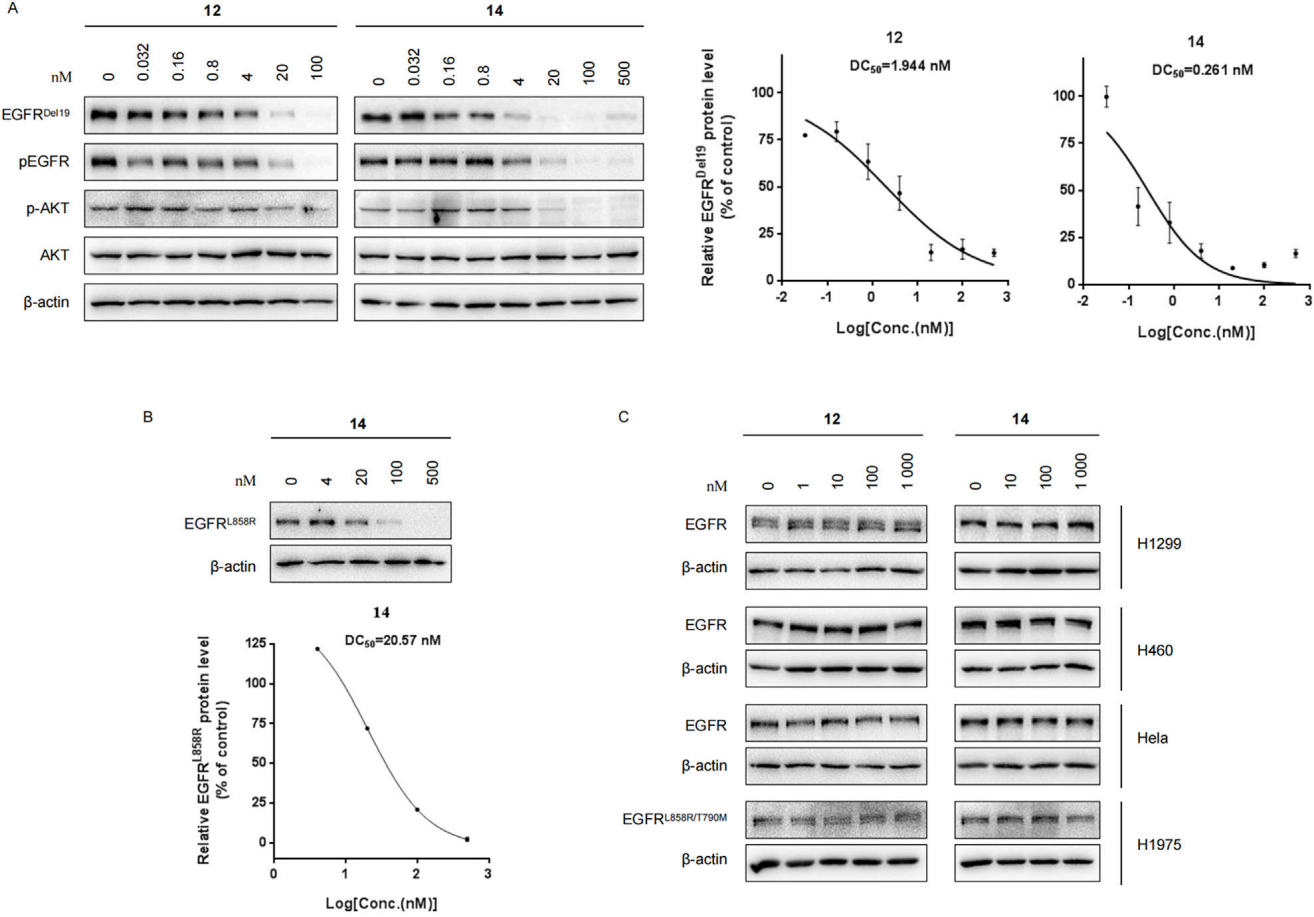
**(A)** Western blots of EGFR^Del19^, pEGFR, AKT, and pAKT in HCC827 cells with compound **12** or **14** for 24 h. The DC_50_ values were calculated using GraphPad Prism. **(B)** Western blots in Ba/F3 cells stably expressing the EGFR^L858R^ mutant with compound **14** for 24 h. **(C)** Immunoblots of EGFR in H1299, H460, HeLa, and H1975 cells treated with compound **12** or **14** for 24 h.

We further evaluated the degradation potencies of compounds **12** and **14**, which were identified as the most potent degraders in the primary study. Both compounds could efficiently degrade EGFR^Del19^ in HCC827 cell lines at 24 h treatment with a DC_50_ value of 1.944 nM for **12** (D_max_ = 85.1%) and 0.261 nM for **14** (D_max_ = 91.2%) (Figure 2A). Concordantly, the levels of both pEGFR and pAKT also markedly reduced upon EGFR degradation. Compound **14** also efficiently degraded EGFR^L858R^ in Ba/F3 cells stably expressing the EGFR^L858R^ mutant with a DC_50_ value of 20.57 nM (Figure 2B). However, neither compound degraded the EGFR^WT^ protein in H1299, H460, and HeLa cell lines nor the EGFR^L858R/T790M^ protein in the H1975 cell line, up to 1 μM (Figure 2C). We also compared the degradation efficacy of compound **14** and **MS154** in both HCC827 and Ba/F3 cell lines. The results indicate that compound **14** showed remarkable improvements in the degradation of EGFR^Del19^ and EGFR^L858R^ than MS154 (Supplementary Figure S2).

**FIGURE 3:**
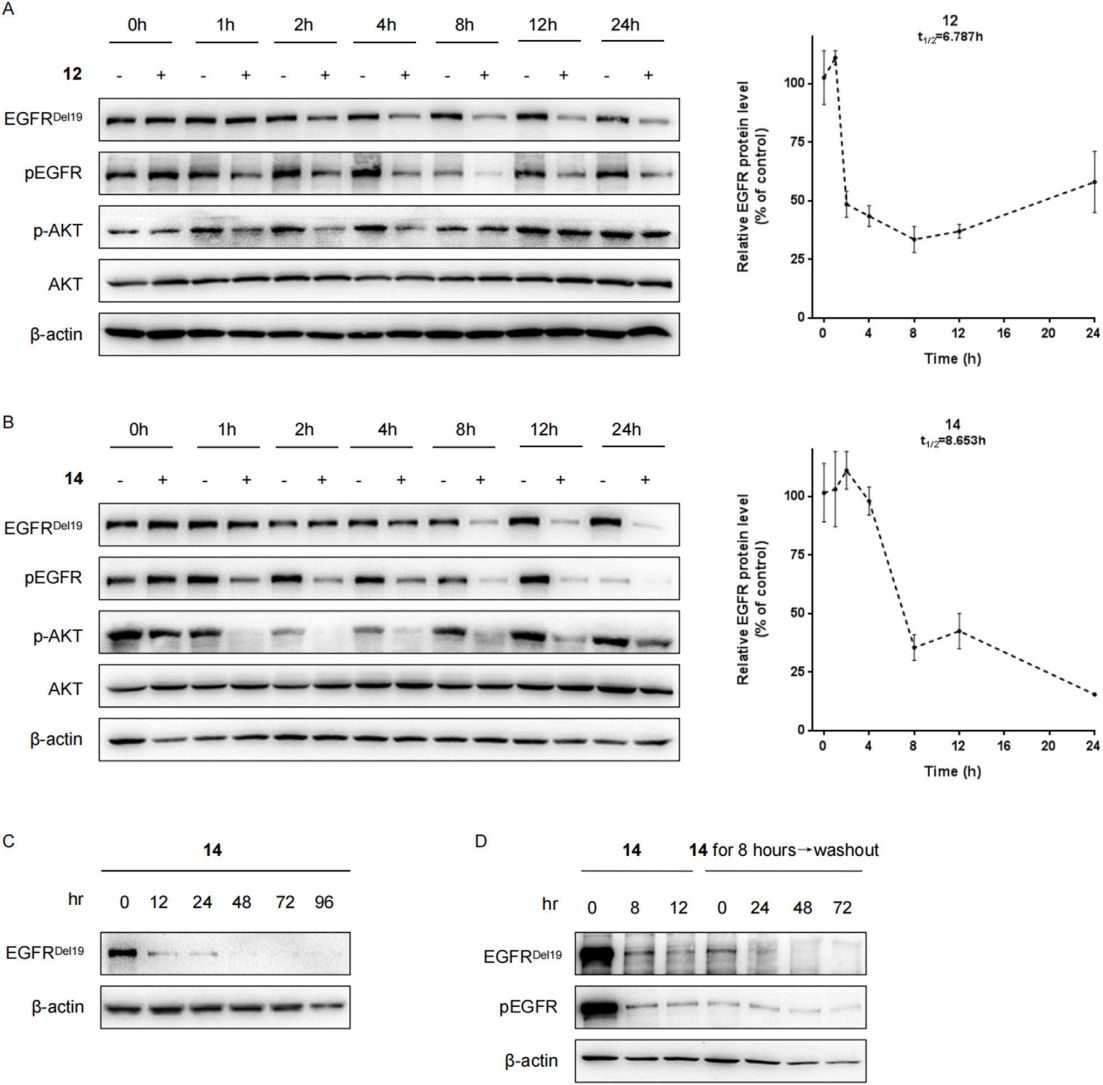
Compounds **12** and **14** time-dependently decreased the EGFR protein in HCC827 cells. **(A)** HCC827 cells were incubated with compound **12 (A)** or **14 (B)** at a final concentration of 10 nM for indicated times (1 h, 2 h, 4 h, 8 h, 12 h, and 24 h). **(C)** HCC827 cells were incubated with compound **14** at a final concentration of 10 nM for indicated times (12 h, 24 h, 48 h, 72 h, and 96 h). **(D)** After pretreatment for 8 h with 10 nM of compound **14**, the media were washed out and replaced with fresh media without compound. After further incubation for the indicated times (24 h, 48 h, and 72 h), HCC827 cells were lysed for Western blot.

Next, the levels of the EGFR^Del19^ protein in HCC827 cells incubated with compound **12** or **14** at a dose of 10 nM at multiple time points were examined (Figure 3). The degradation of EGFR^Del19^ could be observed as early as 2 h after treatment. Compound **14** decreased the half-life of the EGFR^Del19^ protein at 8.6 h and compound **12** at 6.8 h; however, compound **14** induced significantly greater degradation of EGFR, with a maximum degradation (D_max_) of 85% after 24 h of incubation (Figures 3A,B). Meanwhile, compound **14** led to more marked decreases in pEGFR and pAKT compared with compound **12**. In addition, compound **14** mediated a sustained degradation of the EGFR^Del19^ protein over 96 h (Figure 3C). More interestingly, a reduction in the EGFR^Del19^ protein induced by an 8 h-incubation with 10 nM of compound **14** was constantly maintained for over 72 h, even after compound removal (Figure 3D).

**FIGURE 4:**
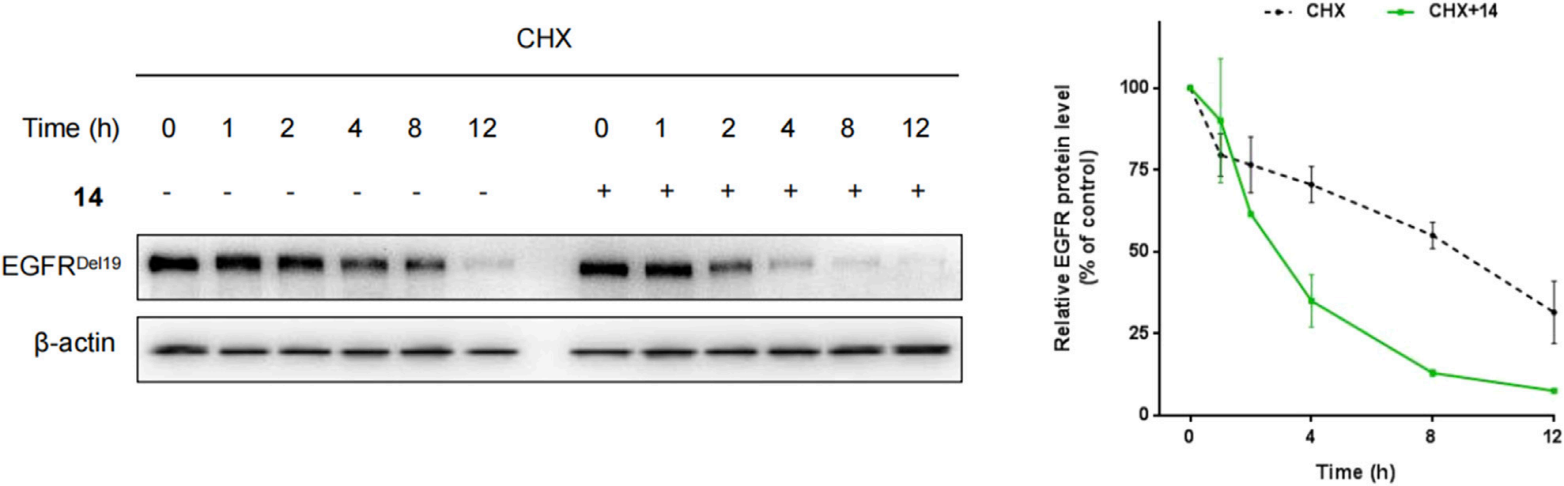
HCC827 cells were incubated with cycloheximide (100 μg/mL) only or cycloheximide (100 μg/mL) with compound **14** (10 nM) for indicated times.

Furthermore, HCC827 cells were incubated with cycloheximide (CHX) with or without compound **14**, and the levels of the EGFR^Del19^ protein were determined. As a result, after 4 h and 8 h of cycloheximide treatment, control DMSO-treated samples still retained approximately 70% and 55% of EGFR proteins, whereas, in the presence of compound **14**, only 34.5% and 12.5% remained (Figure 4).

### Degradation of EGFR^Del19^ using the ubiquitination and proteasome-dependent system

**FIGURE 5:**
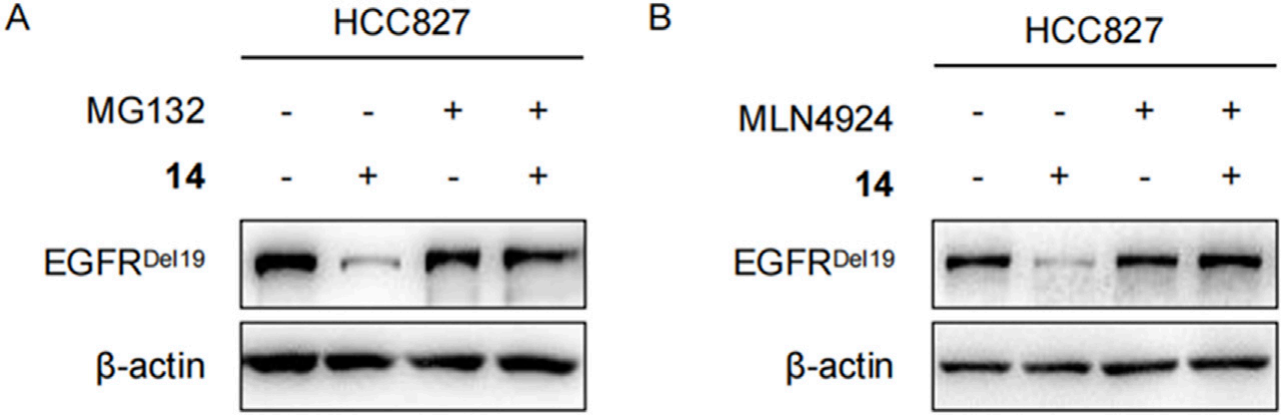
Compound **14** degraded EGFR via proteasome in HCC827 cells. Immunoblots of EGFR in HCC827 cells pretreated with DMSO, 2 μM of MG132 **(A)**, and 5 μM of MLN4924 **(B)** for 2 h and then treated with 10 nM of **14** for 8 h.

To clarify whether these degradations were dependent on E3 ligase complex recruitment, HCC827 cells were pretreated with either the proteasome inhibitor MG132 or the NEDD8-activating enzyme inhibitor MLN4924 for 2 h and continuously incubated with compound **14** for an additional 8 h. As shown in Figures 5A,B, both MG132 and MLN4924 restored EGFR^Del19^ degradation induced by compound **14**, implying that EGFR degradation by compound **14** was dependent on the ubiquitination and neddylation system.

**FIGURE 6:**
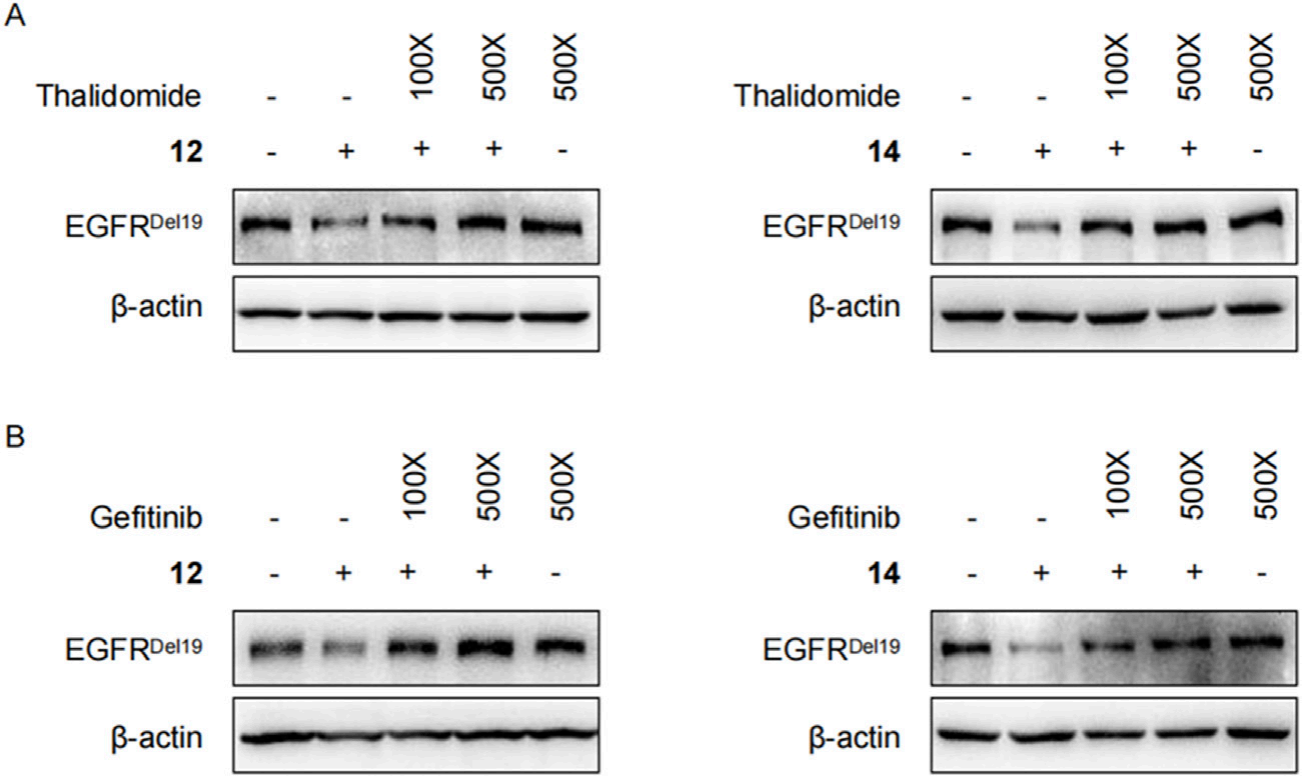
**(A)** Immunoblots of EGFR in HCC827 cells pretreated with thalidomide (1 μM or 5 μM) for 2 h and co-treated with 10 nM of compound **12** (left panel) or **14** (right panel) for 4 h. **(B)** Immunoblots of EGFR in HCC827 cells pretreated with gefitinib (1 μM or 5 μM) for 2 h and co-treated with 10 nM of compound **12** (left panel) or **14** (right panel) for 4 h.

Moreover, competitive binding experiments were conducted with either the CRBN ligand thalidomide (Figure 6A) or the EGFR ligand gefitinib (Figure 6B). Both thalidomide and gefitinib could completely rescue the EGFR^Del19^ reduction induced by compound **12** or **14** at 500 times the degrader dose, whereas a slight decrease in EGFR^Del19^ was still observed at 100 times the dose. These results suggested that compound **14** directly targeted EGFR^Del19^ and mediated EGFR^Del19^ degradation through the CRBN-dependent mechanism.

### Evaluation of cell viability of compounds

**FIGURE 7:**
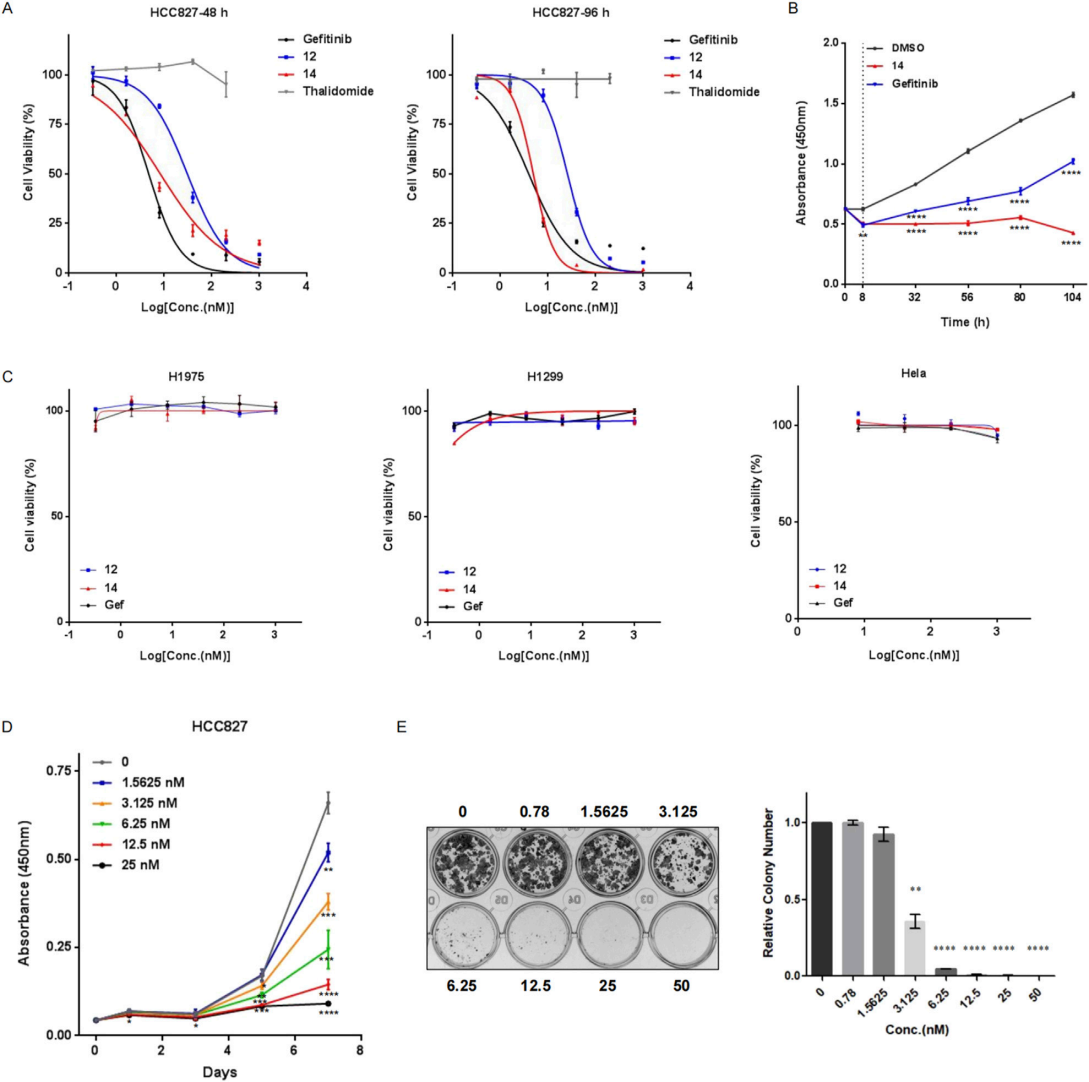
Compound 14 effectively inhibited cell viability of HCC827. **(A)** HCC827 cells were treated with various concentrations of compound **12**, compound **14**, gefitinib, or thalidomide for 48 h (left panel) or 96 h (right panel). **(B)** HCC827 cells were pretreated with compound **14** or gefitinib at 10 nM for 8 h, rinsed with PBS, and cultured for additional indicated times (24 h, 48 h, 72 h, and 96 h). Cell proliferation was measured using CCK-8. **(C)** H1975, H1299, and HeLa cells were treated with compound **12**, compound **14**, or gefitinib for 96 h. Proliferation **(D)** and colony-forming abilities **(E)** of HCC827 cells with different concentrations of compound **14** were examined. Results are presented as the mean ± SD of three independent experiments. *p*-values were calculated using the Student’s t-test (*p < 0.05; **p < 0.01; ***p < 0.001; ****p < 0.0001)

To study the biological effects of the decrease in PROTAC-mediated EGFR^Del19^ on cancer cell survival, HCC827 cells were treated with compound **12**, compound **14**, gefitinib, or thalidomide for 48 h or 96 h, and the number of viable cells was calculated using CCK-8 (Figure 7). With a 48 h-incubation, the IC_50_ values of compound **12**, compound **14**, and gefitinib were approximately 30.68 ± 6.15 nM, 8.29 ± 3.31 nM, and 4.74 ± 1.19 nM, respectively (Figure 7A). With a 96 h-incubation, the IC_50_ values of compound **12**, compound **14**, and gefitinib were approximately 25.64 ± 3.8 nM, 4.91 ± 0.78 nM, and 3.93 ± 1.4 nM, respectively (Figure 7A). In particular, compound **14** exhibited cytotoxic activity similar to that of gefitinib. Thalidomide had no influence on the viability of HCC827 cells up to 200 nM. To explore the prolonged efficacy, HCC827 cells were treated with 10 nM of compound **14** or gefitinib for 8 h, washed with PBS, and cultured with fresh media for an additional 24, 48, 72, or 96 h. Intriguingly, gefitinib-pretreated cells gradually continued to proliferate after the removal of compounds, whereas compound **14**-pretreated cells almost completely stopped proliferating (Figure 7B), illustrating the distinct pharmacological mechanisms of compound **14** and gefitinib.

In addition, compound **12**, compound **14,** and gefitinib did not affect the viability of H1299, HeLa, and H1975 cells up to 1 μM, suggesting high specificity of PROTACs toward the EGFR^Del19^ mutant (Figure 7C). Concordantly, compound **14** markedly attenuated the proliferation of HCC827 cells compared with control vehicle-treated cells (Figure 7D) and formed fewer and smaller colonies in HCC827 cells than the control in colony formation assays (Figure 7E).

### Degradation of EGFR^Del19^ induces cell apoptosis in HCC827 cells

**FIGURE 8:**
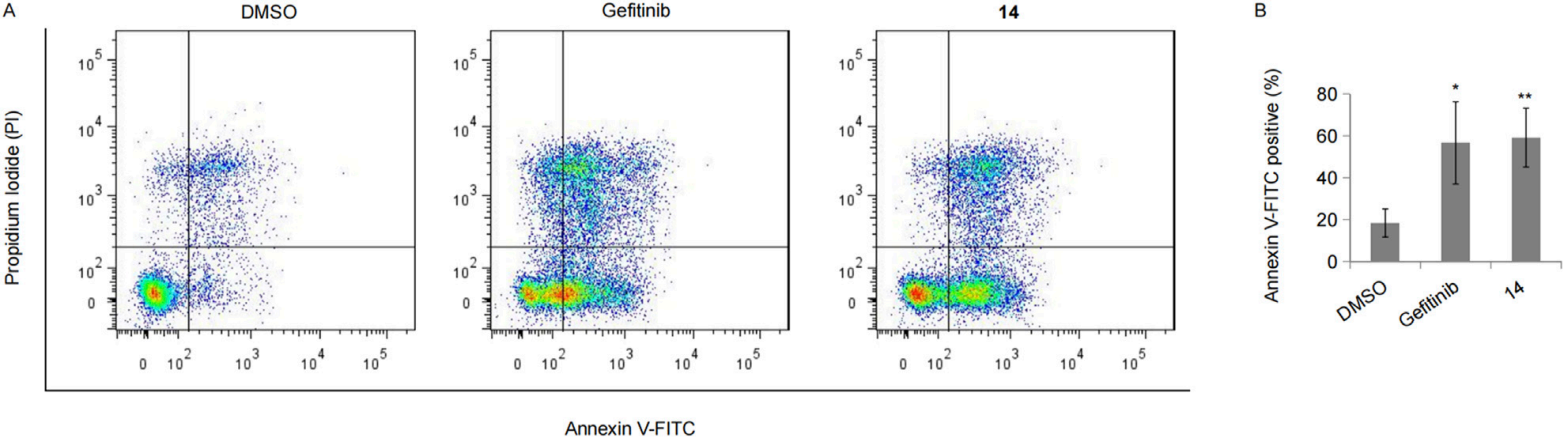
Compound 14 induced cell apoptosis. **(A)** HCC827 cells incubated with 10 nM of gefitinib or compound **14** for 48 h were double-stained with Annexin V-FITC and PI, and the representative result was shown. **(B)** The experiment was repeated three times, and data represented the average percentage of apoptotic cells (*p < 0.05 and **p < 0.01; Student’s t-test).

In the above study, numerous apoptotic cells were observed under the microscope following treatment with compound **14**. Hence, we performed FACS analysis to further calculate the apoptotic cell population upon treatment with compound **14** or gefitinib in HCC827 cells. As expected, both gefitinib and compound **14** led to at least a two-fold increase in the number of apoptotic cells, and there was little difference between compound **14** and gefitinib (Figures 8A,B).

### *In vivo* anti-tumor activity of the compound

**FIGURE 9:**
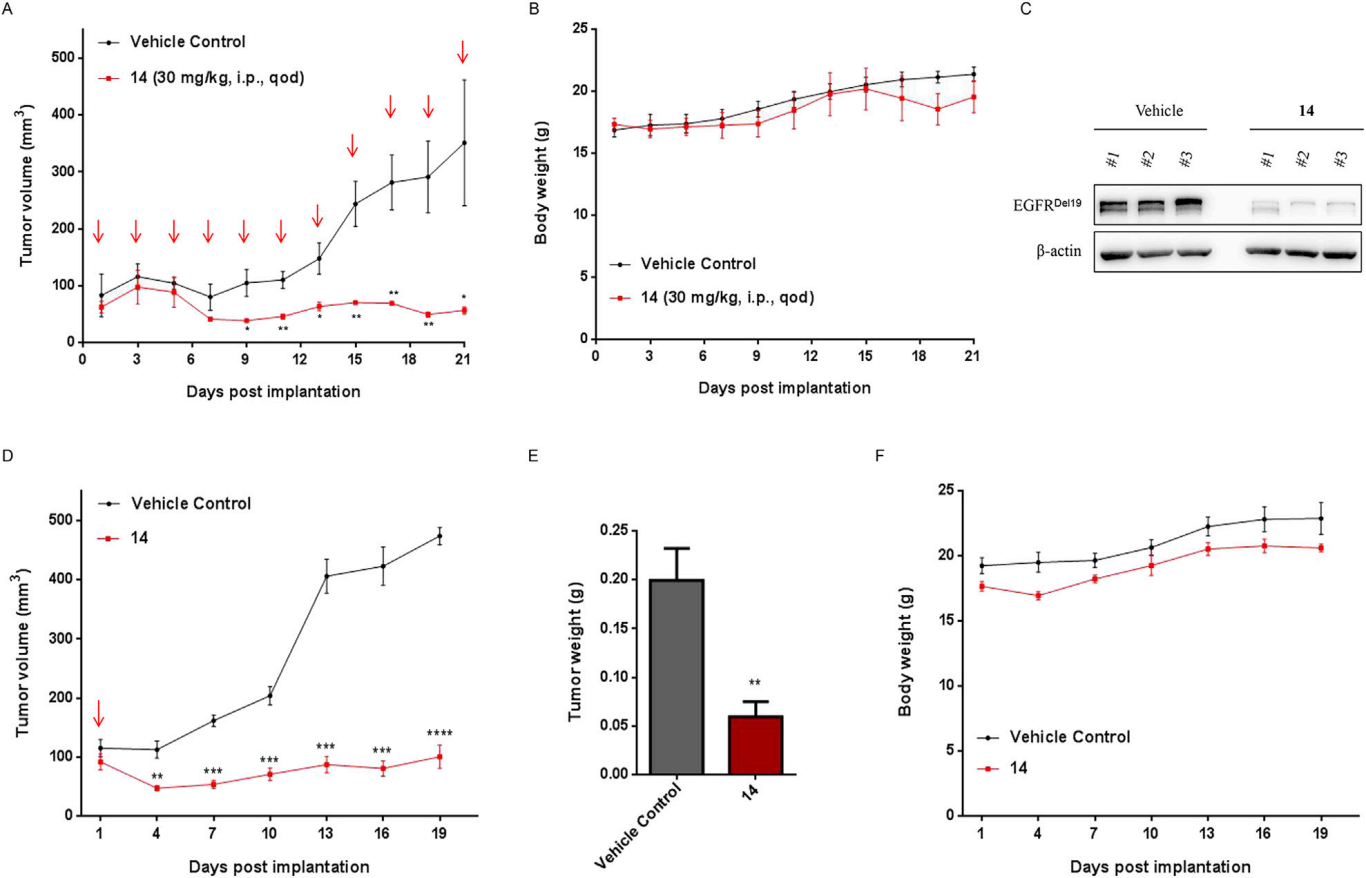
Compound 14 inhibited the growth of human tumor xenografts. Nude mice bearing established HCC827 human tumor xenograft were dosed with either vehicle or compound **14** (30 mg/kg) via an intraperitoneal injection every other day for 22 days. Tumor volumes **(A)** and mouse weights **(B)** were measured every other day and plotted against time. Each point represented the mean ± SD of three tumors. **(C)** The indicated proteins of dissected tumors were detected using Western blot. **(D)** Nude mice bearing established HCC827 human tumor xenograft were administered a one-time intraperitoneal injection of compound 14 (30 mg/kg). Tumor volumes, tumor weights **(E)**, and mouse weights **(F)** were measured every 3 days and plotted against time. Each point represented the mean ± SD of three tumors. *p < 0.05; **p < 0.01; ***p < 0.001; ****p < 0.0001.

We evaluated the efficacy of compound **14** to inhibit the growth of HCC827 cells using a xenograft immunodeficient mouse model. As shown in Figure 9A, compound **14** (30 mg/kg) markedly suppressed the growth of implanted tumors. The mean volume of HCC827 tumors treated with compound **14** (56.58 ± 10.91 mm^3^) was noticeably decreased compared with that of control vehicle mice (350.86 ± 191.41 mm^3^) (Figure 9A, *p* < 0.05). Meanwhile, compound **14** effectively degraded the EGFR^Del19^ protein *in vivo* (Figure 9C). Furthermore, mice-bearing HCC827 xenografts were administered a one-time intraperitoneal injection of compound **14** at 30 mg/kg. Intriguingly, as shown in Figures 9D,E, one dose of compound **14** led to a mean tumor volume inhibition of 79% (P = 5.4E-05 versus vehicle control) 18 days after treatment, indicating the long-term effect for this compound. None of the mice showed signs of wasting (Figures 9B,F).

## Conclusions

In this study, a series of EGFR degraders were designed using computational methods based on gefitinib and thalidomide. Compound **14** was identified as a potent and selective degrader against both EGFR^Del19^ and EGFR^L858R^ proteins, with DC_50_ values of 0.26 nM and 20.57 nM, respectively. No significant degradation of the wild-type EGFR was observed, even at a concentration of 1 μM. Compound **14** significantly reduced the viability of HCC827 cells while having no effect on EGFR wild-type cells, exhibiting a 96-h IC_50_ value of 4.91 nM, similar to the cytotoxic activity of gefitinib. Notably, cells pretreated with gefitinib showed a mild attenuation of proliferation after the compound was removed, whereas cells pretreated with compound **14** completely ceased proliferation, indicating the long-lasting effects of compound **14**. The degradation of EGFR^Del19^ by compound **14** was confirmed to be dependent on ubiquitination and the proteasome pathway. In the HCC827 cell-derived xenograft model, compound **14** also demonstrated excellent anti-tumor efficacy *in vivo*. With a relatively low molecular weight of 814.32 Da and impressive *in vitro* and *in vivo* efficacy, compound 14 may serve as a lead for further development of drug-like EGFR degraders.

## Data Availability

The original contributions presented in the study are included in the article/Supplementary Material; further inquiries can be directed to the corresponding author.
