# Supplementary material for: Targeted degradation of EGFR 19Del by PROTACs suppresses tumor growth in non-small-cell lung cancer
Source: Front Pharmacol. 2025 Oct 24;16:1604661. doi: 10.3389/fphar.2025.1604661 (PMC12592173; doi:10.3389/fphar.2025.1604661)

**The supporting information of the article entitled:**

**Targeted Degradation of EGFR 19Del by Protac Suppresses Tumor Growth in Non-Small cell Lung Cancer**

*Lianhua Piao^1, 2, *^, Ying Gao^2, *^, Yangyang Su^1^, Qihui Li^2^, Xiaofeng Yuan^3^, Wanzhou Zhao^4^, Janne Kulpakko^5^, Pei-Chieng Cha^6^, Shan Chang^1^, Ren Kong^1, 2, #^*

^1^Institute of Bioinformatics and Medical Engineering, Jiangsu University of Technology, Changzhou, Jiangsu 213001, P.R.China

^2^ Primary Biotechnology Co., Ltd., Suzhou 215125, P.R.China

^3^ Department of Orthopaedics, The Third Affiliated Hospital of SooChow University, Changzhou, Jiangsu 213000, P.R.China

^4^ The Nanjing Han & Zaenker Cancer Institute (NHZCI), OG Pharmaceuticals, Nanjing, Jiangsu 210036, P.R.China

^5^ Aqsens Health Oy, Itäinen Pitkäkatu 4B, 20520 Turku, Finland

^6^ Department of Genomic Medicine, Research Institute, National Cerebral and Cardiovascular Center, Suita, Japan

*Correspondence to: Ren Kong

Ren Kong - rkong@jsut.edu.cn

**Content of Supporting Information**

1. Supplementary Table S1………………………………………………………………… 2

2. Supplementary Figure S1……………………………………………………………….. 3

3. Supplementary Figure S2……………………………………………………………….. 4

4. Supplementary Figure S3……………………………………………………………….. 4

5. Supplementary schemes for the synthesis of compounds .……………………………… 5

6. NMR traces of final compounds ..……………………………………………………… 10

7. HPLC traces of final compounds…………………………………………………………18

8. NMR traces and LCMS data for **MS154** purchased from MCE ……………………….. 26

**1. Supplementary Table S1**. Distance distribution of tethering atoms from Top 100 poses.

| Distance (Å) | Distribution Percentage (%) |
| --- | --- |
| 0-1 | 0 |
| 1-2 | 0 |
| 2-3 | 0 |
| 3-4 | 0 |
| 4-5 | 0 |
| 5-6 | 0 |
| 6-7 | 0 |
| 7-8 | 1 |
| 8-9 | 5 |
| 9-10 | 1 |
| 10-11 | 0 |
| 11-12 | 4 |
| 12-13 | 8 |
| 13-14 | 8 |
| 14-15 | 10 |
| 15-16 | 12 |
| 16-17 | 17 |
| 17-18 | 18 |
| 18-19 | 5 |
| 19-20 | 9 |
| 20-21 | 2 |
| 21-22 | 0 |
| 22-23 | 0 |
| 23-24 | 0 |
| 24-25 | 0 |
| 25-26 | 0 |
| 26-27 | 0 |
| 27-28 | 0 |
| 28-29 | 0 |
| 29-30 | 0 |

**2. Supplementary Figure S1**. Docking poses of EGFR and CRBN generated by “site constraint docking” mode of CoDock. The top 20 poses are shown for clarity. CRBN is represented by cartoon model in green, and the poses of EGFR kinase domain are represented by cartoon model in different colors.


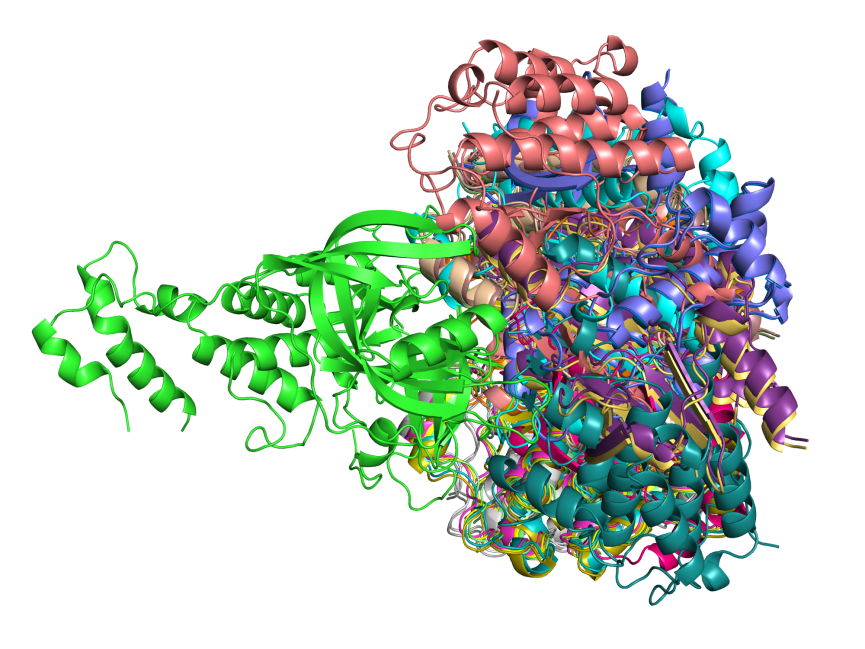


1. **Supplementary Figure S2**. Expression of EGFR^Del19^ in HCC827 cells with the treatment of compound 1-15 at various concentrations for 24 h.


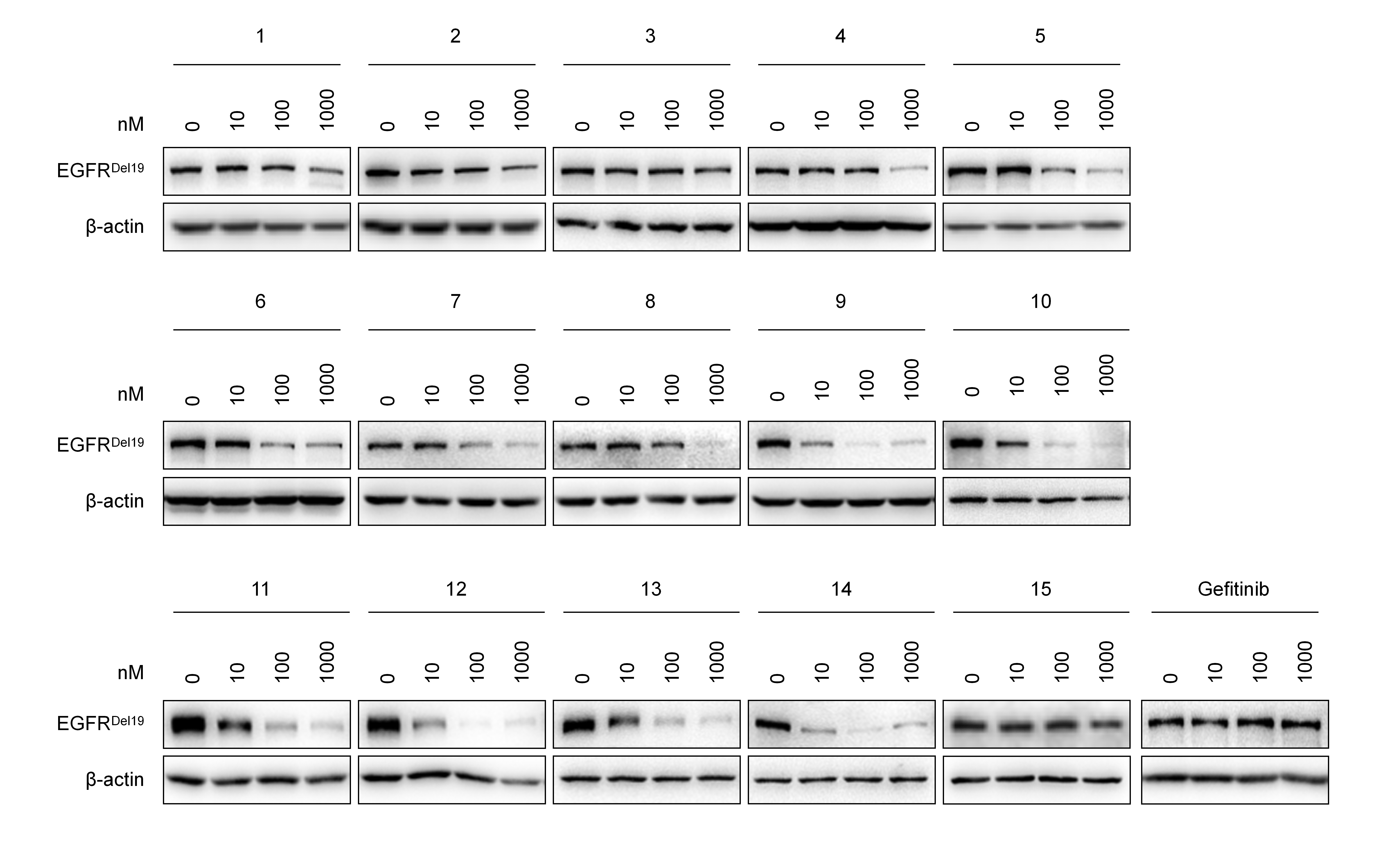


1. **Supplementary Figure S3**.Western blots in HCC827 cells and Ba/F3 cells stably expressing EGFR^L858R^ mutant with compound **14** or **MS154** for 24 h.


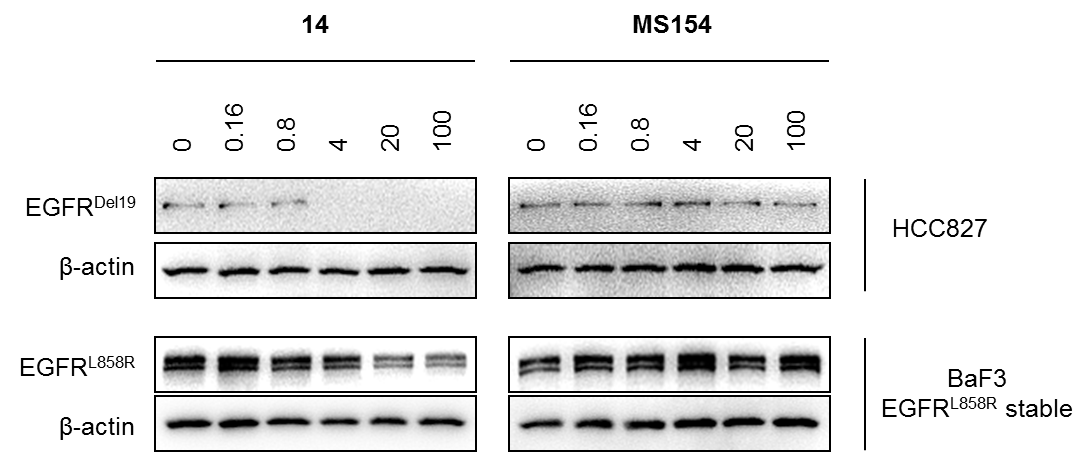


1. **Supplementary scheme for the synthesis of compounds**

**Supplementary Scheme S1.**Synthesis of compounds 1-3, 5, 7, 8. Reagents and reaction conditions: (a) for compounds 1a-3a: Na_2_CO_3_ in DMF 75°C 5h, 39%~42% yield; for compounds 5a,7a: HATU, DIPEA in DMSO, 50°C, overnight, 13%~14% yield; for compound 8a: DIPEA,DMSO,85°C, 3h, yield 37%; (b) pyridine hydrochloride, 150°C, 6h, 69% yield; (c) K_2_CO_3_, KI in DMF, 85°C, 15h, 15%~25% yield.

**Supplementary Scheme S2.**Synthesis of compound 4. Reagents and reaction conditions: (I) 1. DIPEA in DMF 90°C, 6h, 86% yield; 2. HCl/Dioxane, 0°C -r.t., 2h, 76% yield; (II) DIPEA in DMSO, 135°C, 2h, 17% yield; (III) Na_2_CO_3_ in DMF, 75°C, 2h, 9% yield.

**Supplementary Scheme S3.**Synthesis of compound 6. Reagents and reaction conditions: (I) DIPEA in DMSO, 90, 16h, 98% yield; (II) HCl/EtOH, 0°C~r.t., 24h, 60% yield; (III) HATU, DIPEA in DMF, 50°C, 2h, 48% yield; (IV) Na_2_CO_3_ in DMF, 105°C, 2h, 25% yield.

**Supplementary Scheme S4.**Synthesis of compound 9. Reagents and reaction conditions: (I) (COCl)_2_, DMF in DCM, r.t. 2-5h, crude; (II) TEA in DCM, r.t., overnight, 27% yield(two steps) (III) DIPEA, KI in DMF, 85, 15h, 35% yield; (IV) HCl/Dioxane, r.t., 3h, 99% yield; (V) 3-bromopropan-1-ol, K_2_CO_3_ in MeCN, 85°C,15h, 66% yield; (VI) tosyl chloride, triethylamine, 4-dimethylaminopyridine in DCM, r.t., overnight, 86% yield; (VII) Firstly, NaI in acetone, reflux, 3h; Secondly, K_3_PO_4_ in DMF, 55°C, 15h, 12% yield.


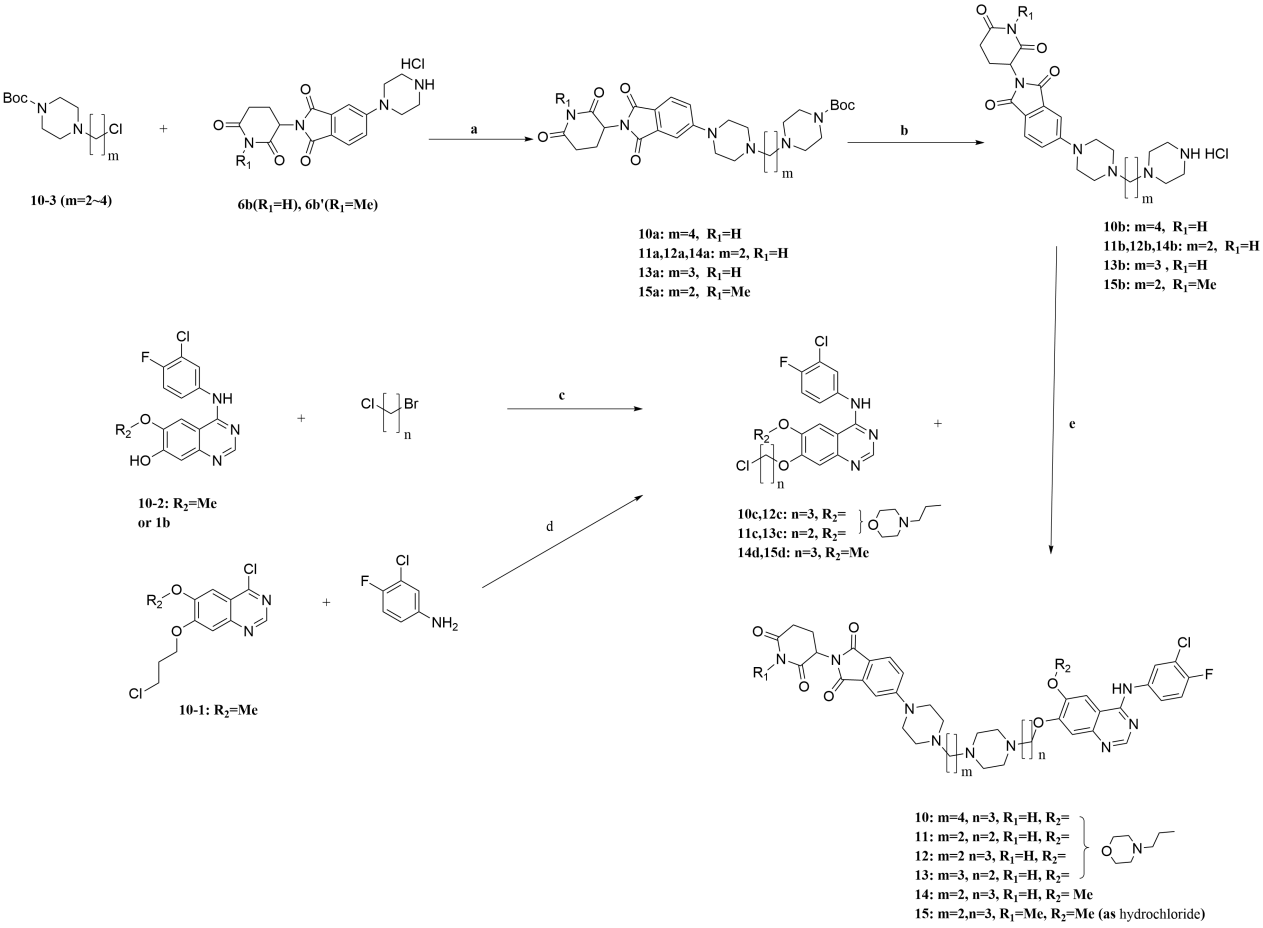


**Supplementary Scheme S5.**Synthesis of compound 10-15. Reagents and reaction conditions: (a) DIPEA in DMF, 85°C, 16h, 20%~53% yield; (b) HCl/Dioxane, 0°C-r.t., 1-2h, 60%~74% yield; (c) K_2_CO_3_ in DMF, 50°C, 16h, 60%~64% yield; (d) 3-chloro-4-fluoroaniline in isopropyl alcohol, 87°C,3h,73% yield; (e) Firstly, NaI in acetone, reflux, 6h; Secondly, K_3_PO_4_ in DMF, 55°C, 16h, 5%~31% yield.

1. **NMR traces of final compounds**

^1^H NMR spectra compound 1


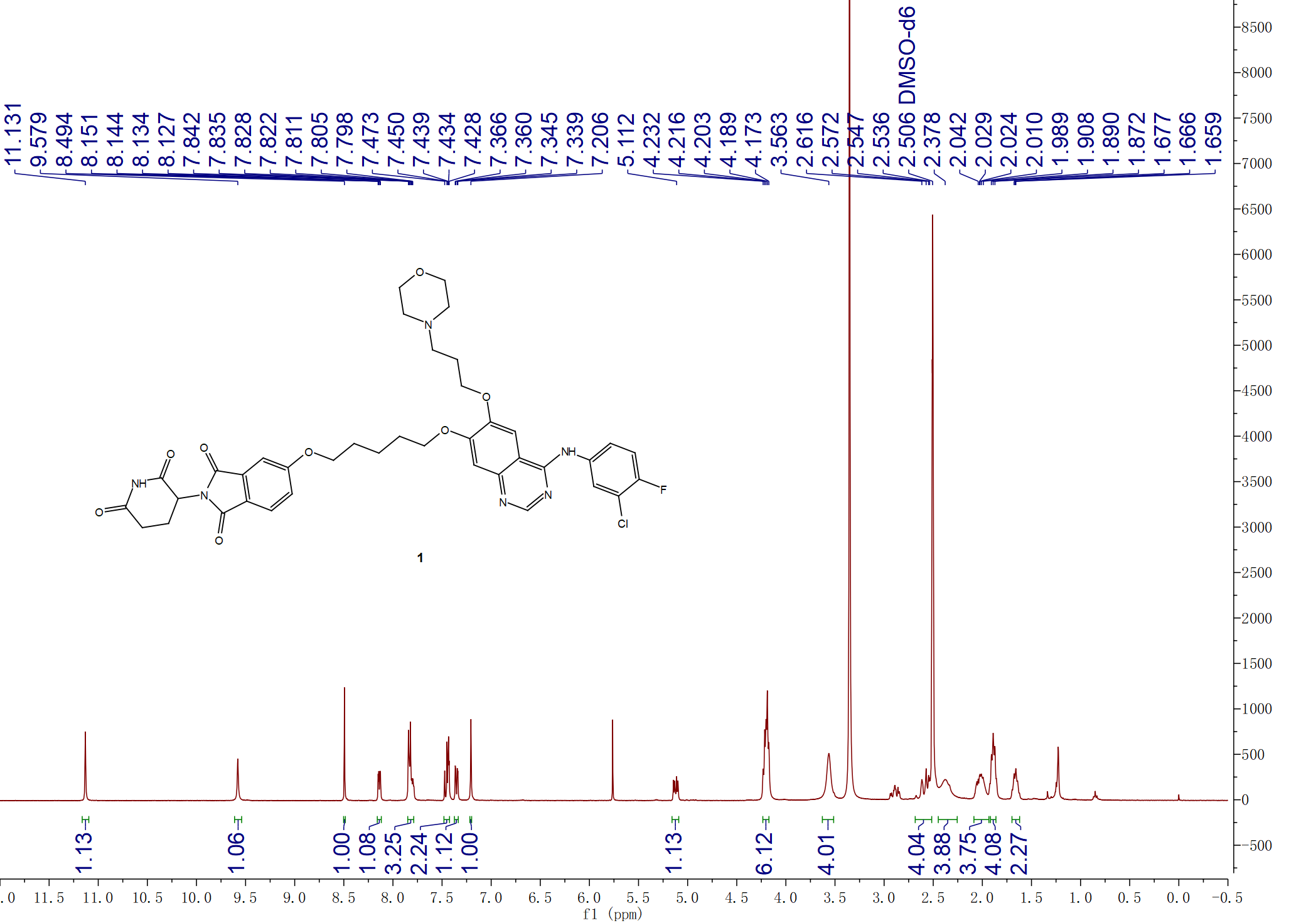


^1^H NMR spectra compound 2


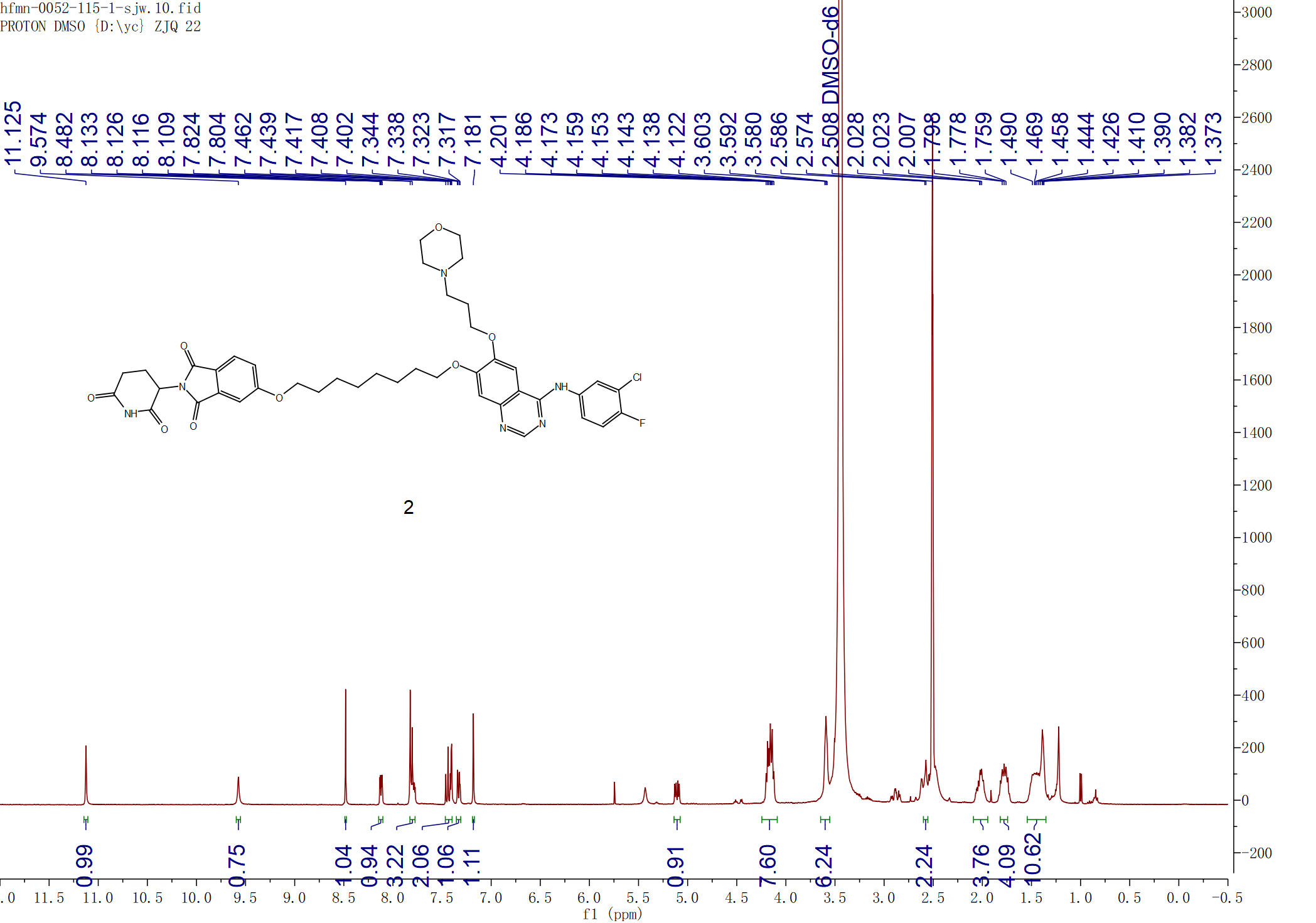


^1^H NMR spectra compound 3


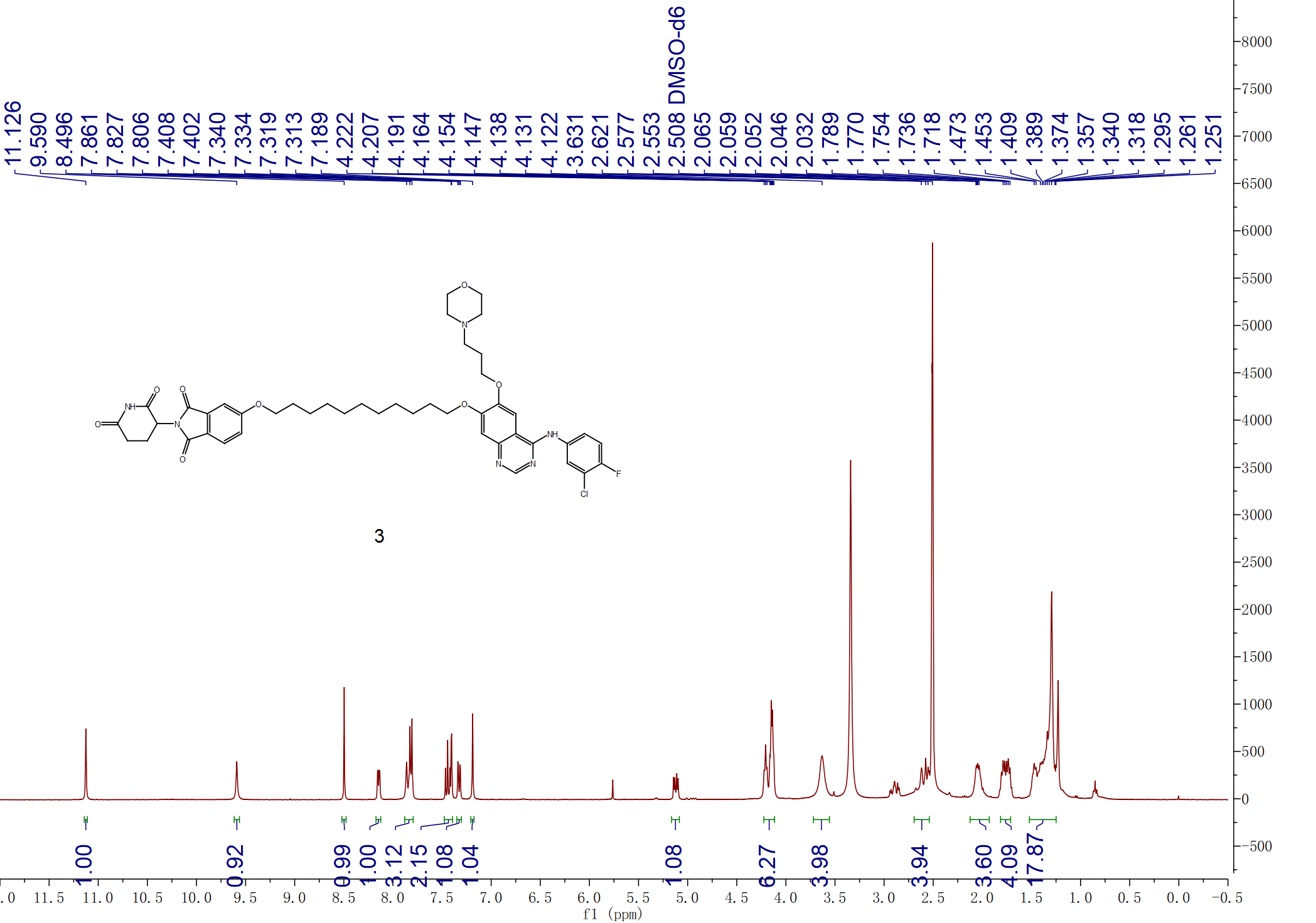


^1^H NMR spectra compound 4


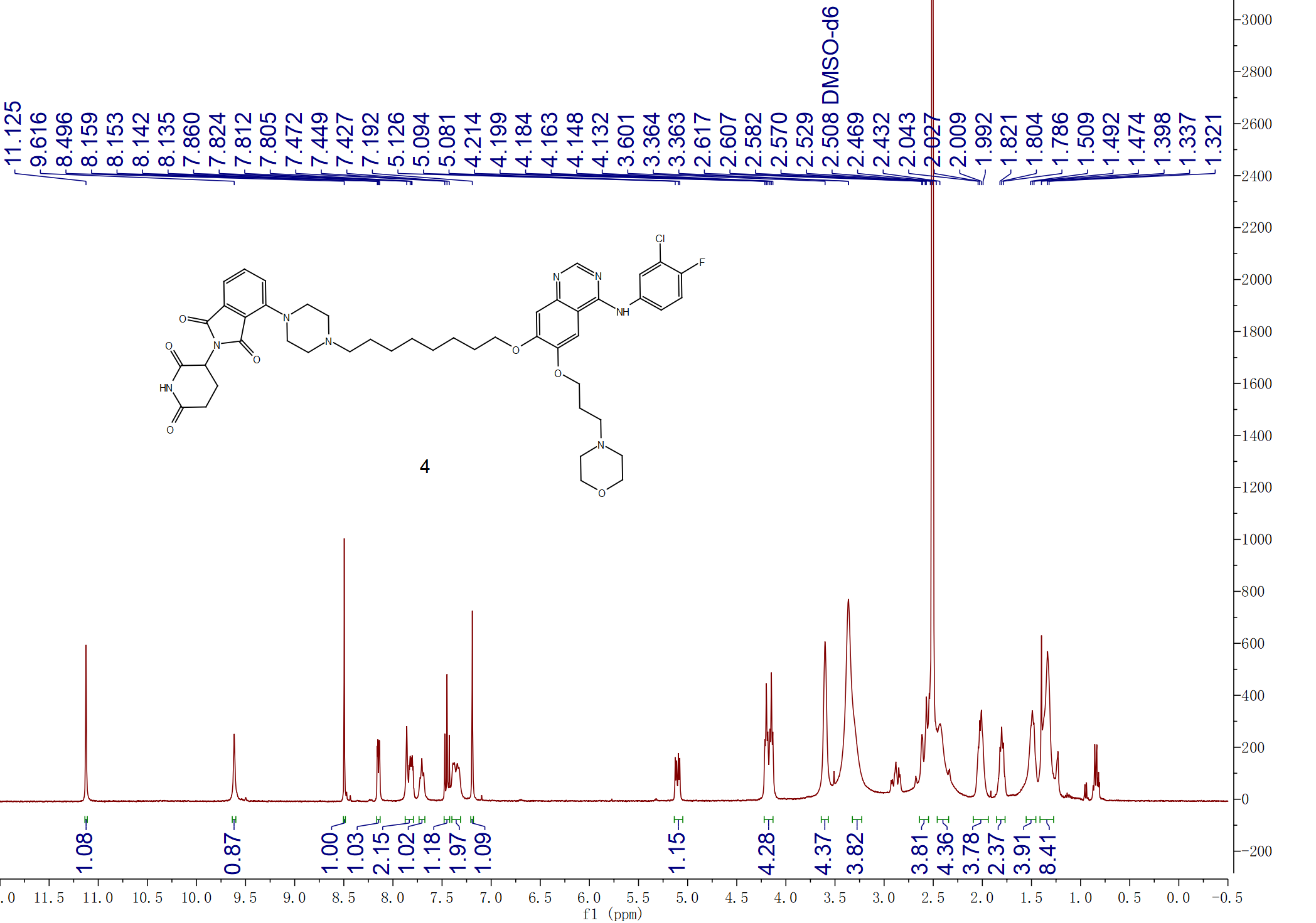


^1^H NMR spectra compound 5


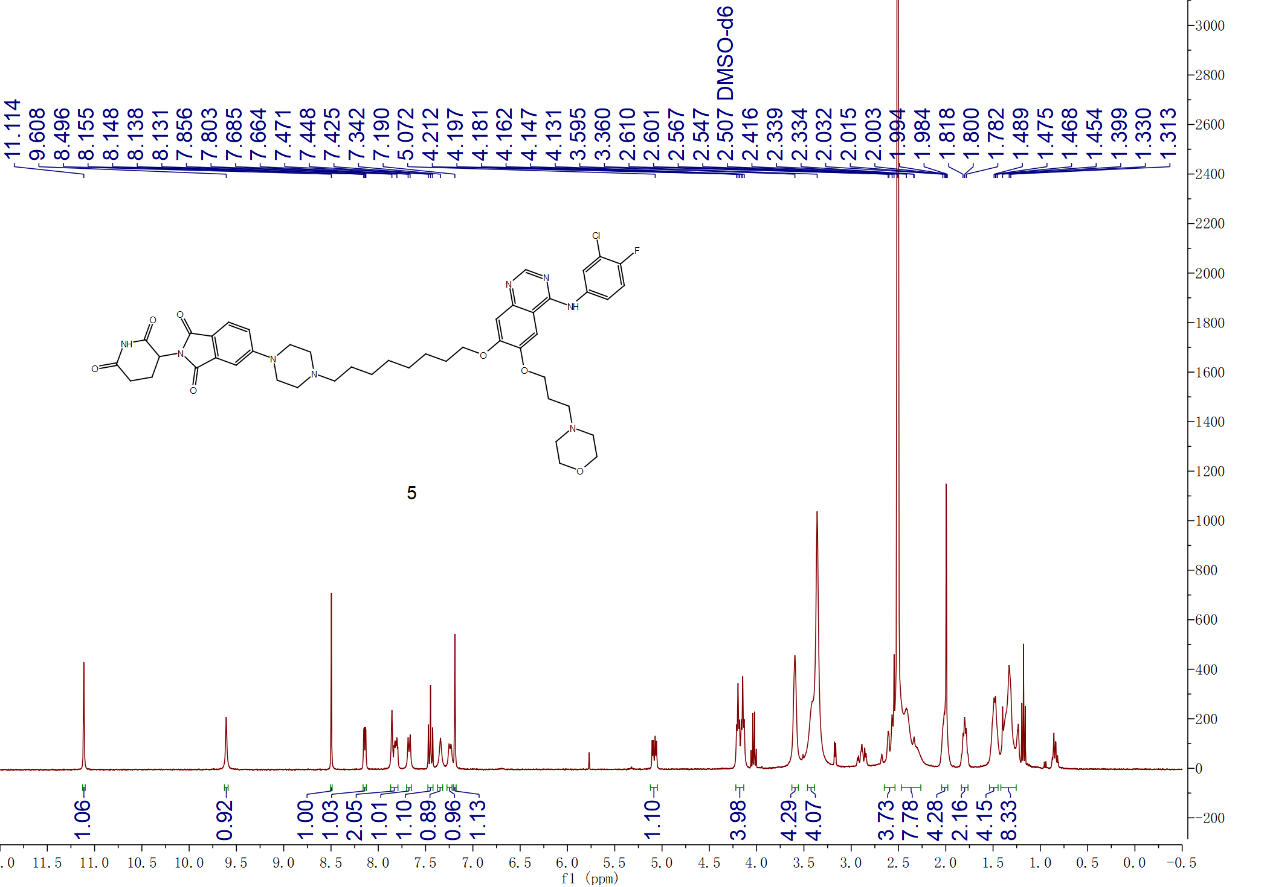


^1^H NMR spectra compound 6


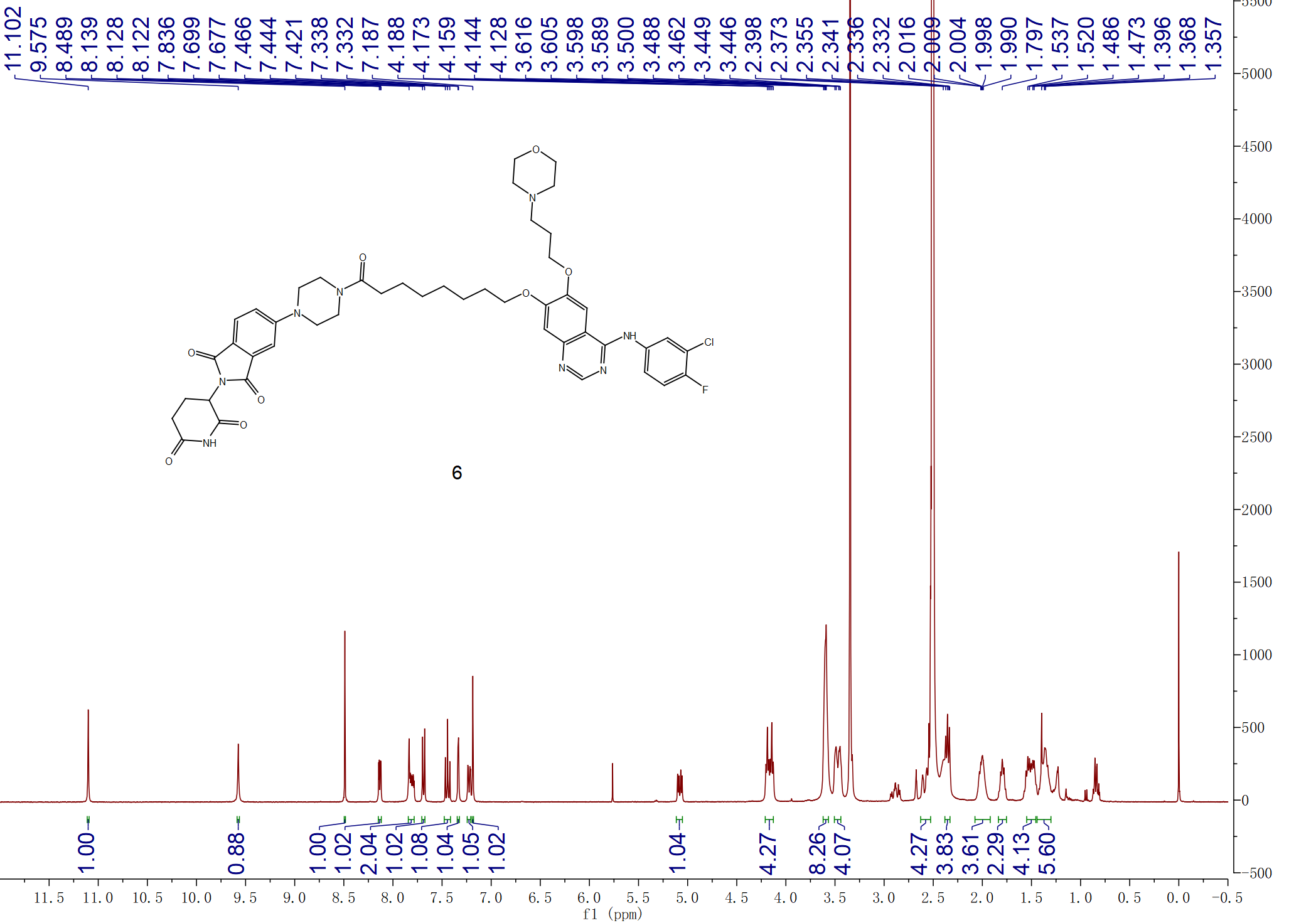


^1^H NMR spectra compound 7


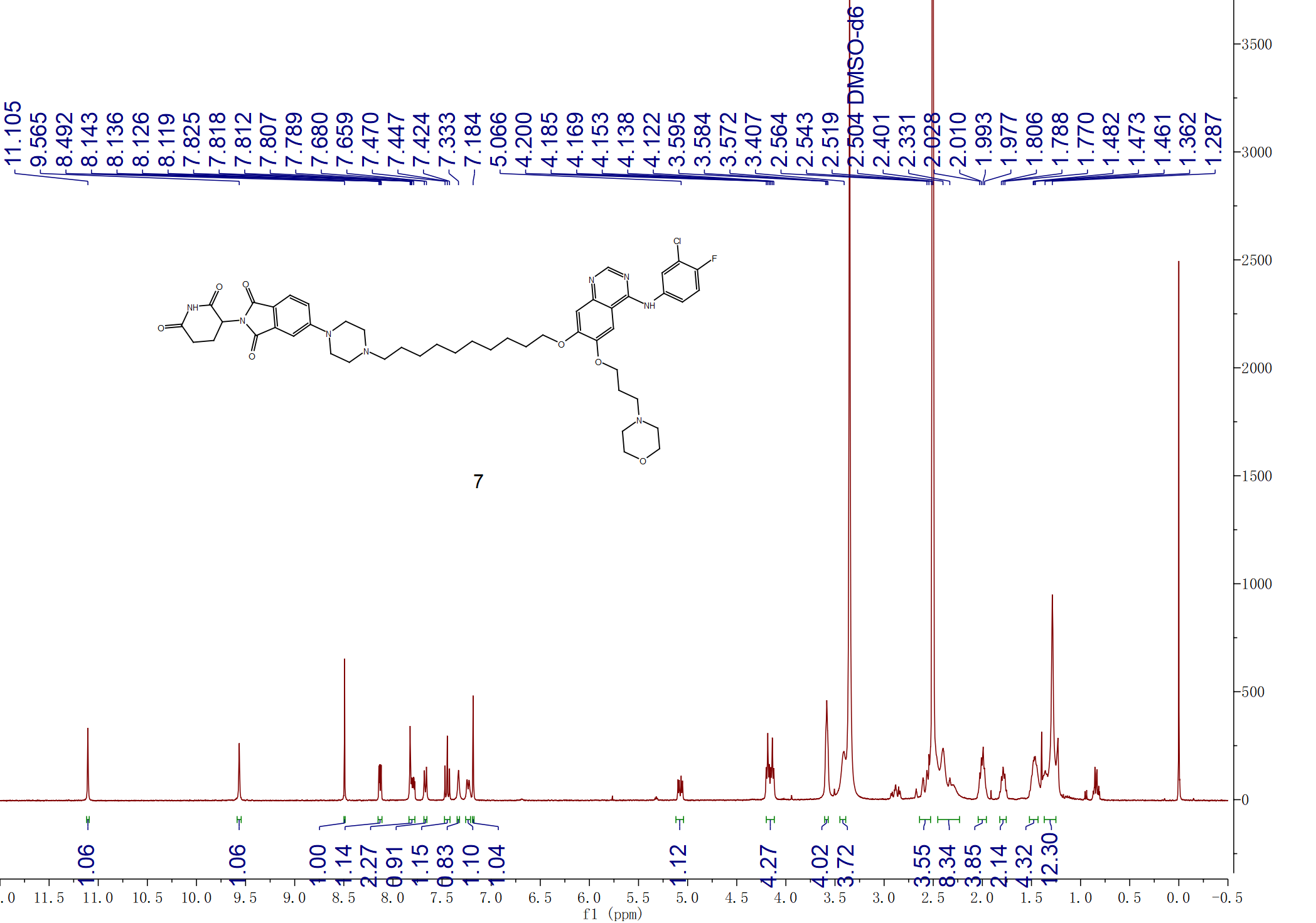


^1^H NMR spectra compound 8


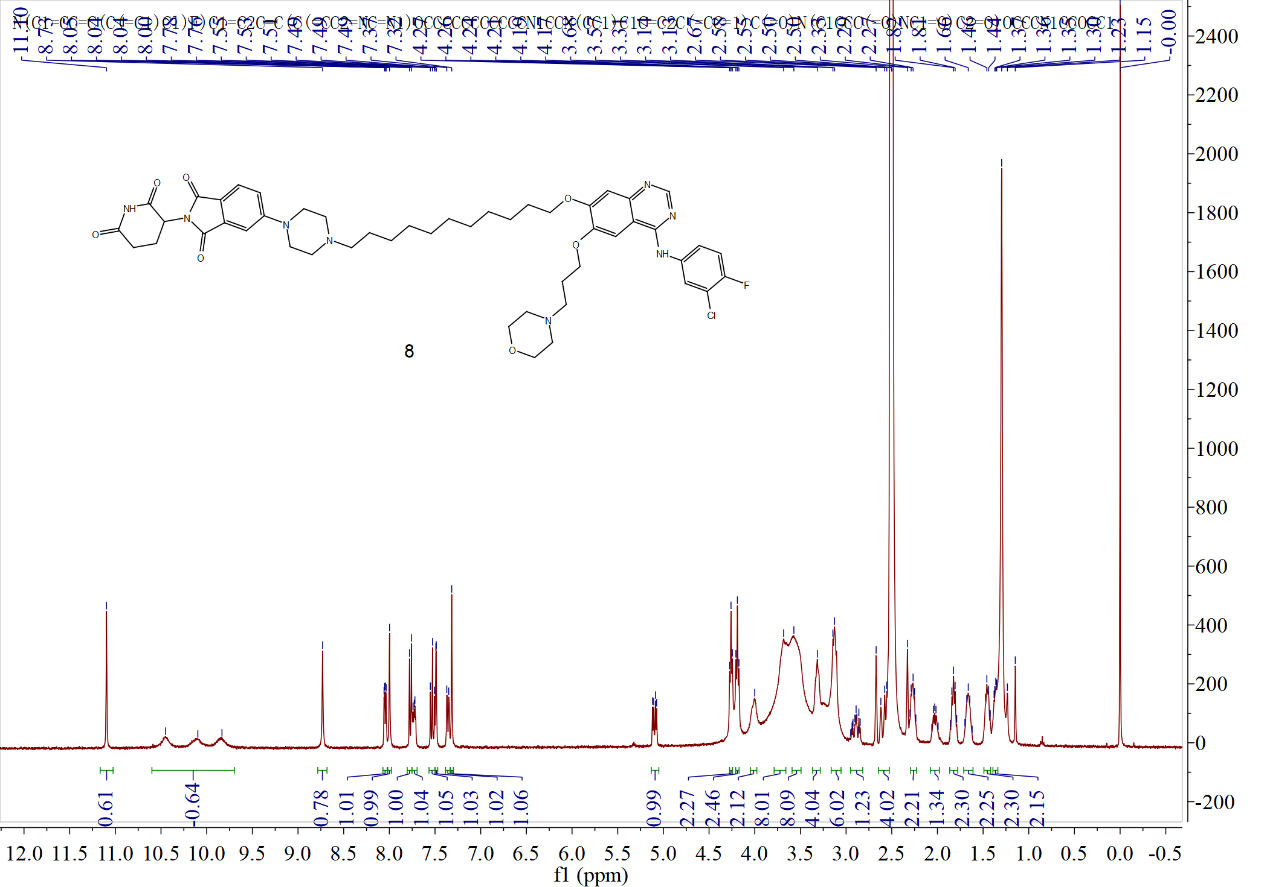


^1^H NMR spectra compound 9


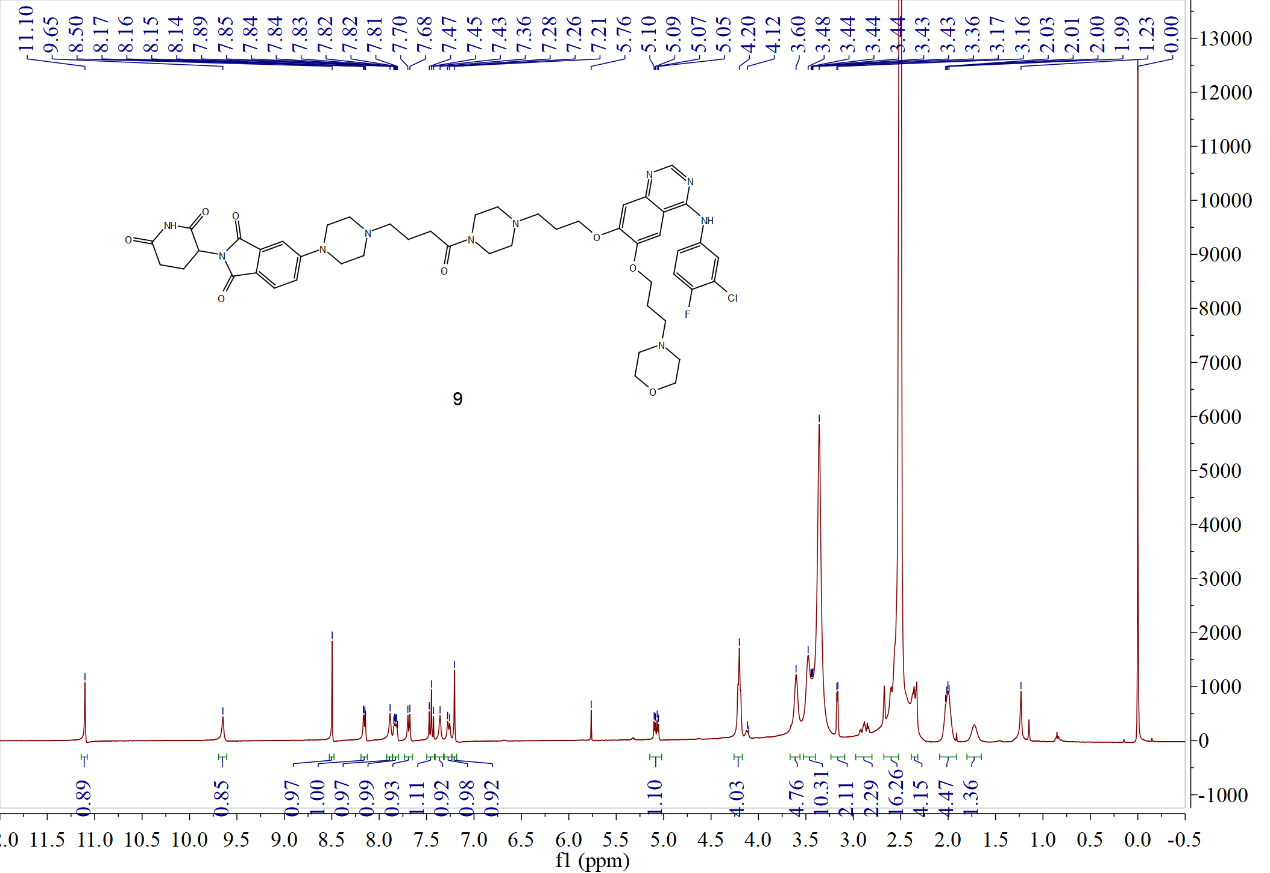


^1^H NMR spectra compound 10


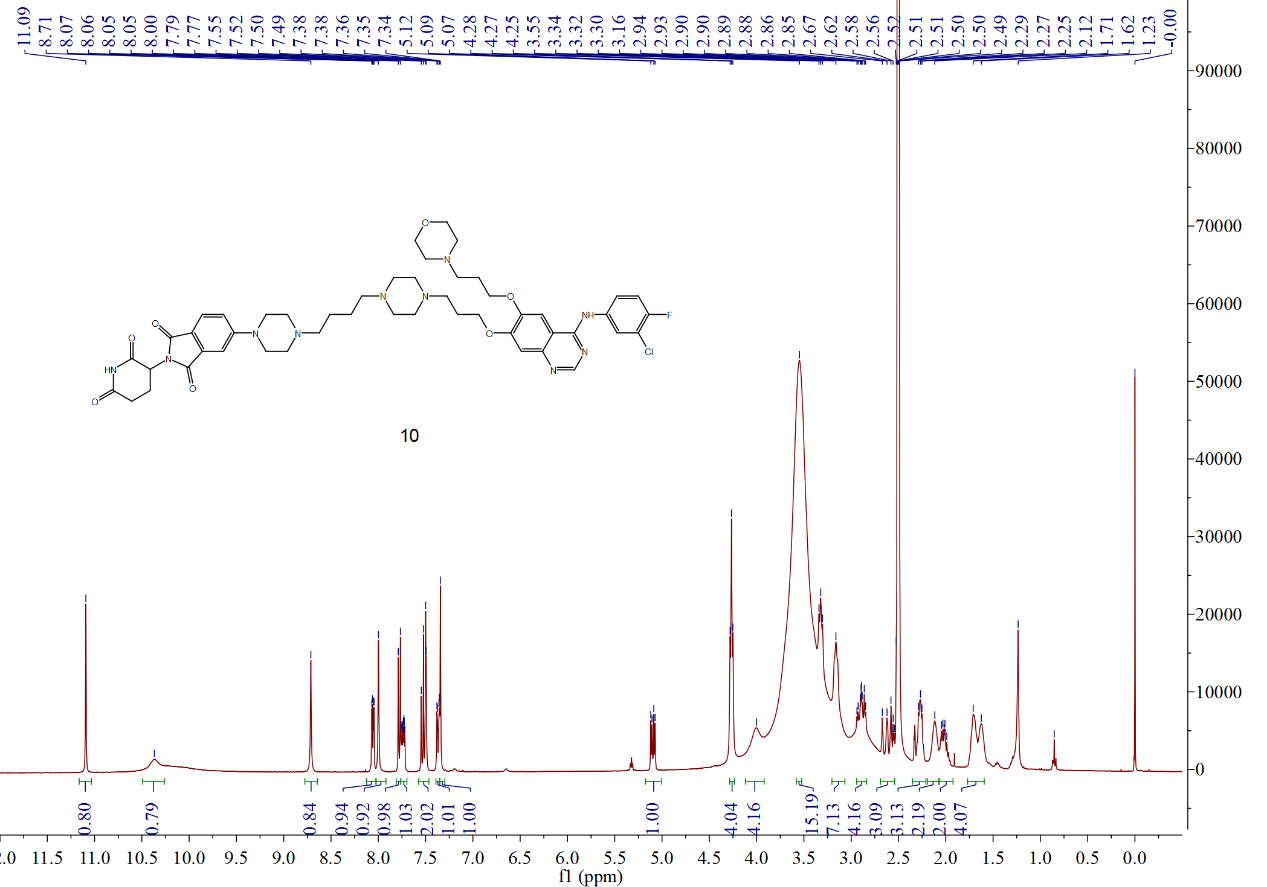


^1^H NMR spectra compound 11


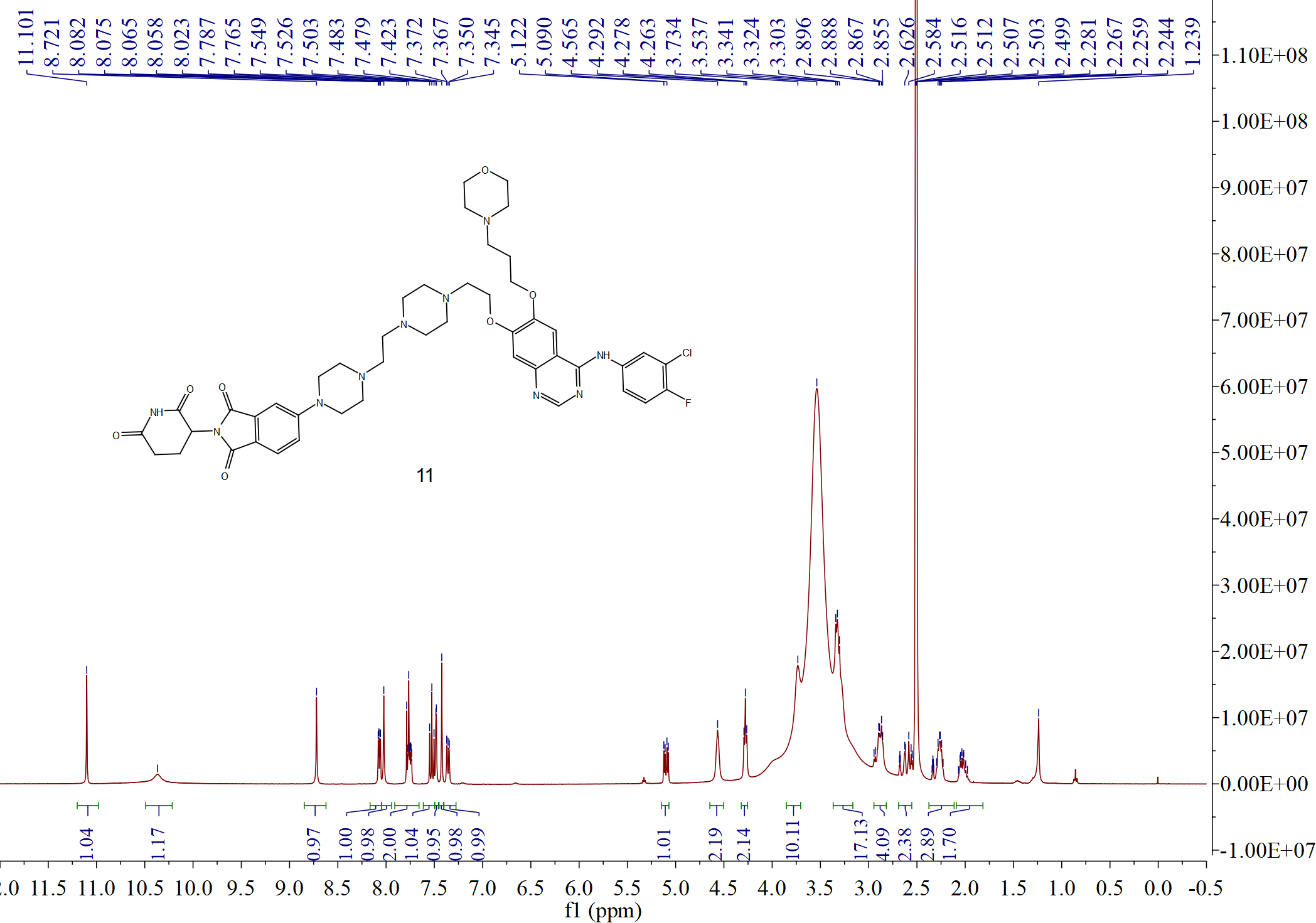


^1^H NMR spectra compound 12


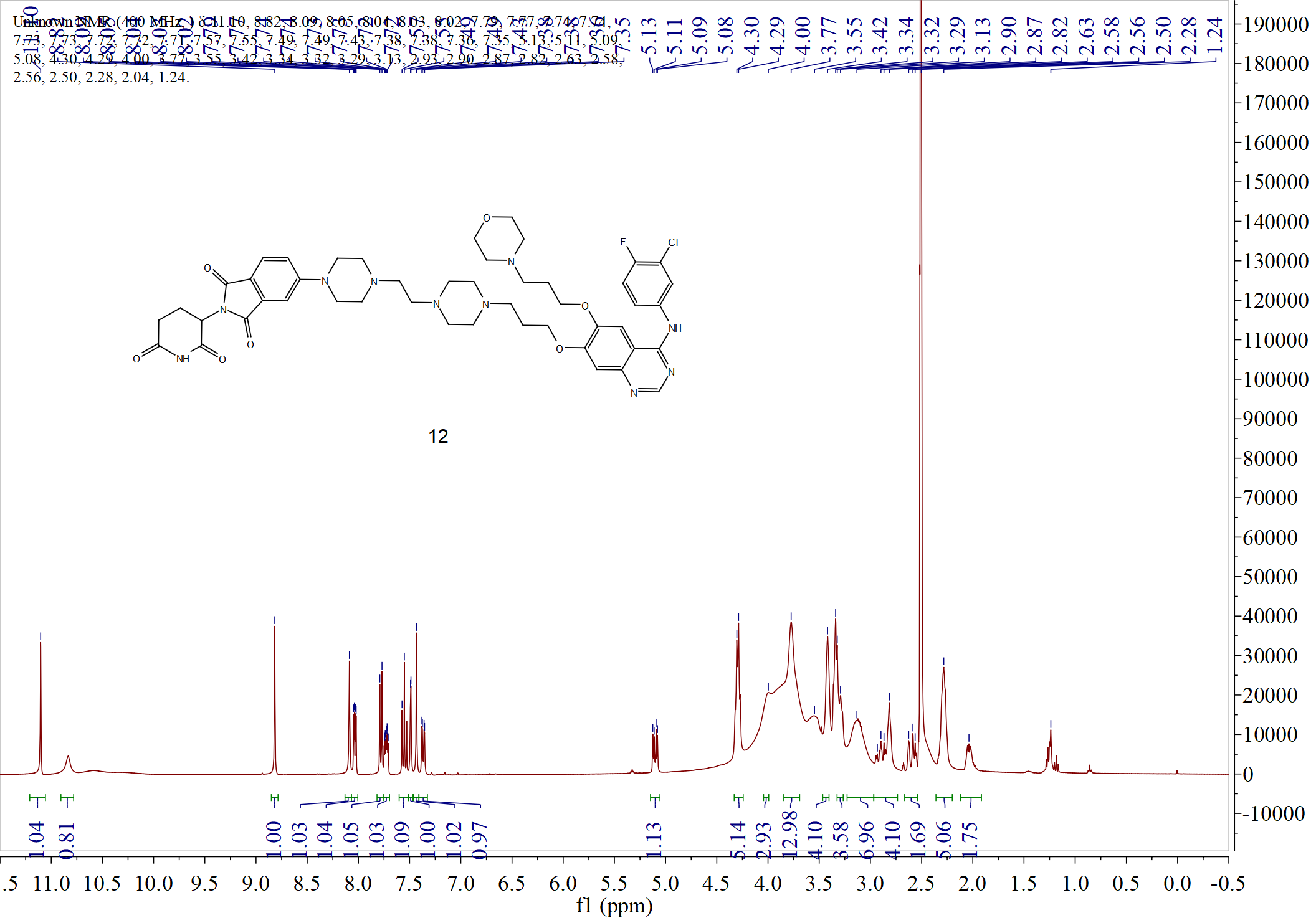


^1^H NMR spectra compound 13


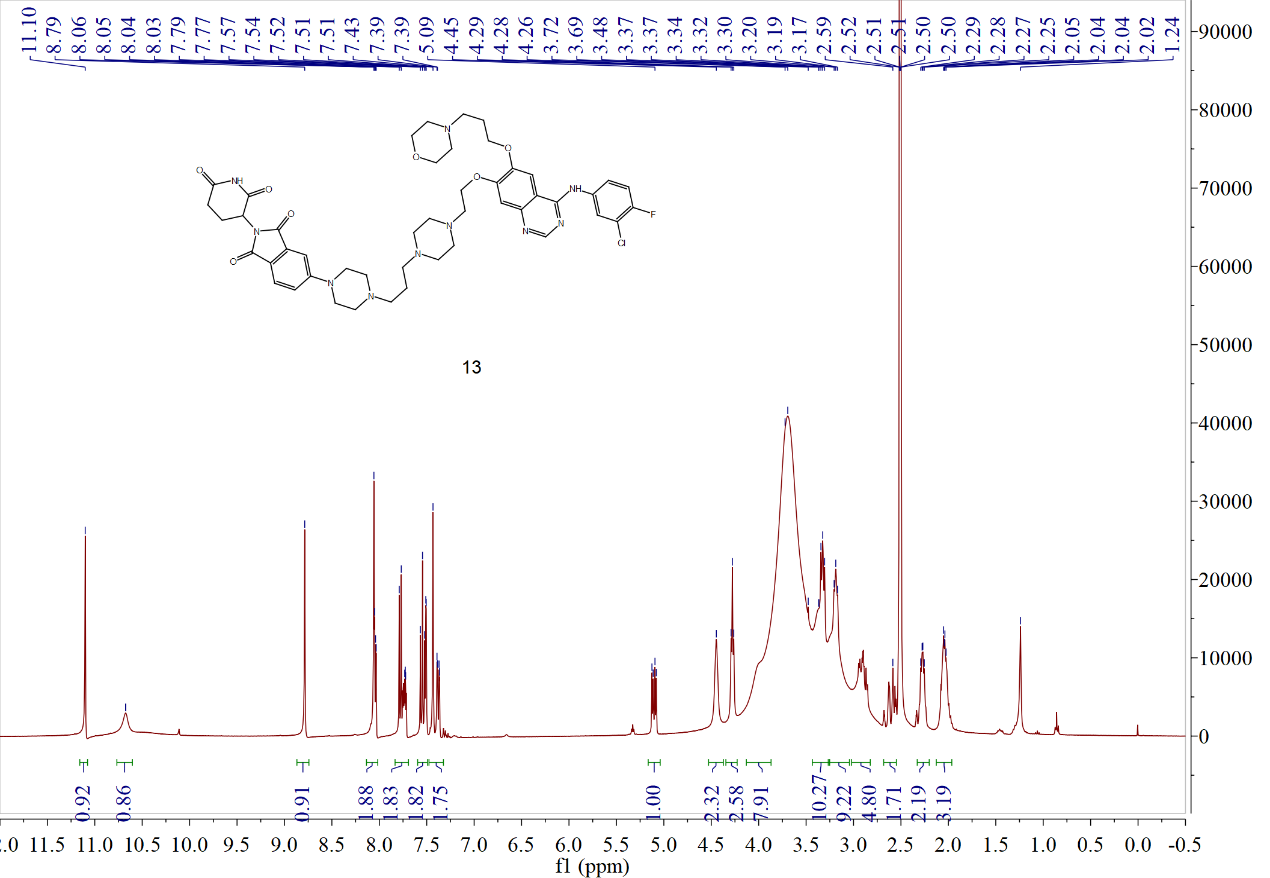


^1^H NMR spectra compound 14


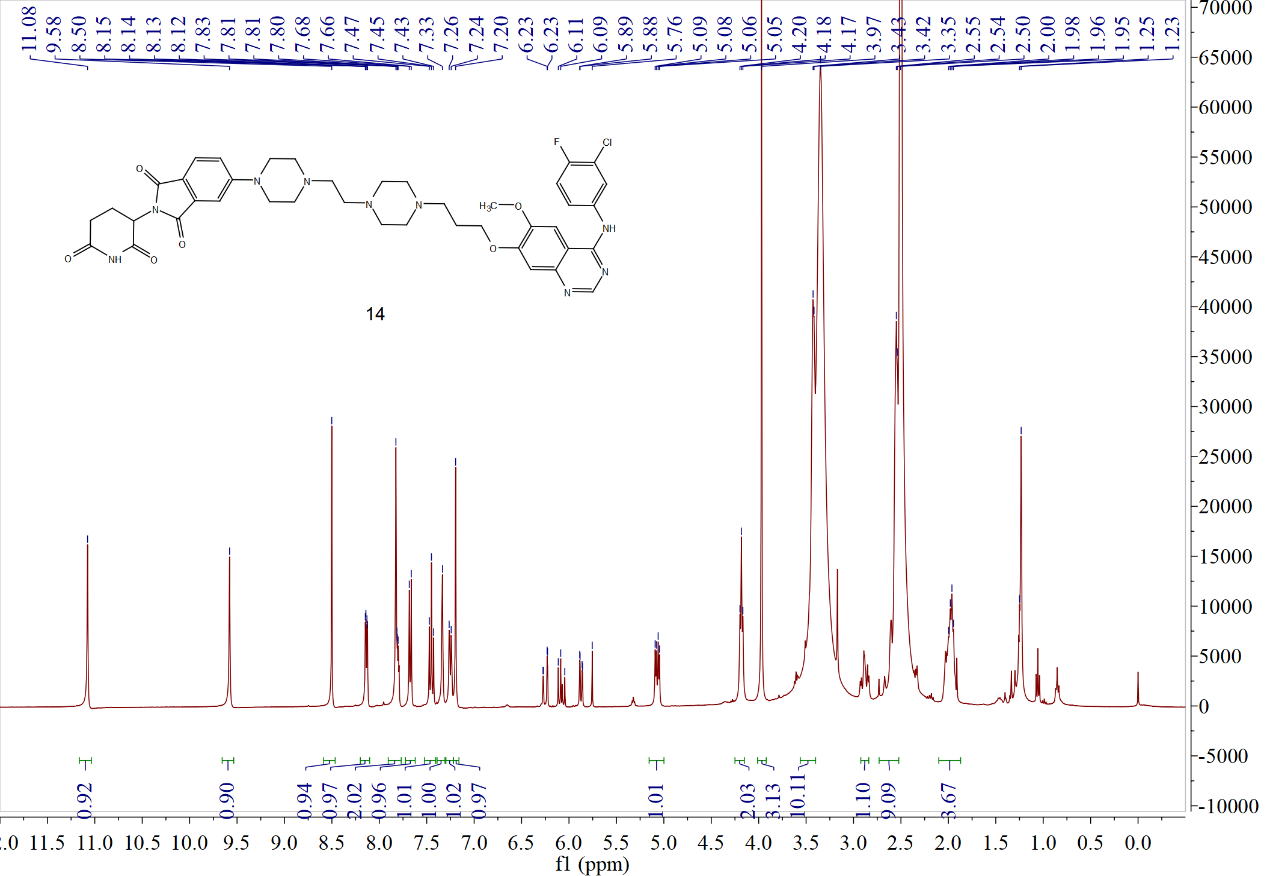


^1^H NMR spectra compound 15


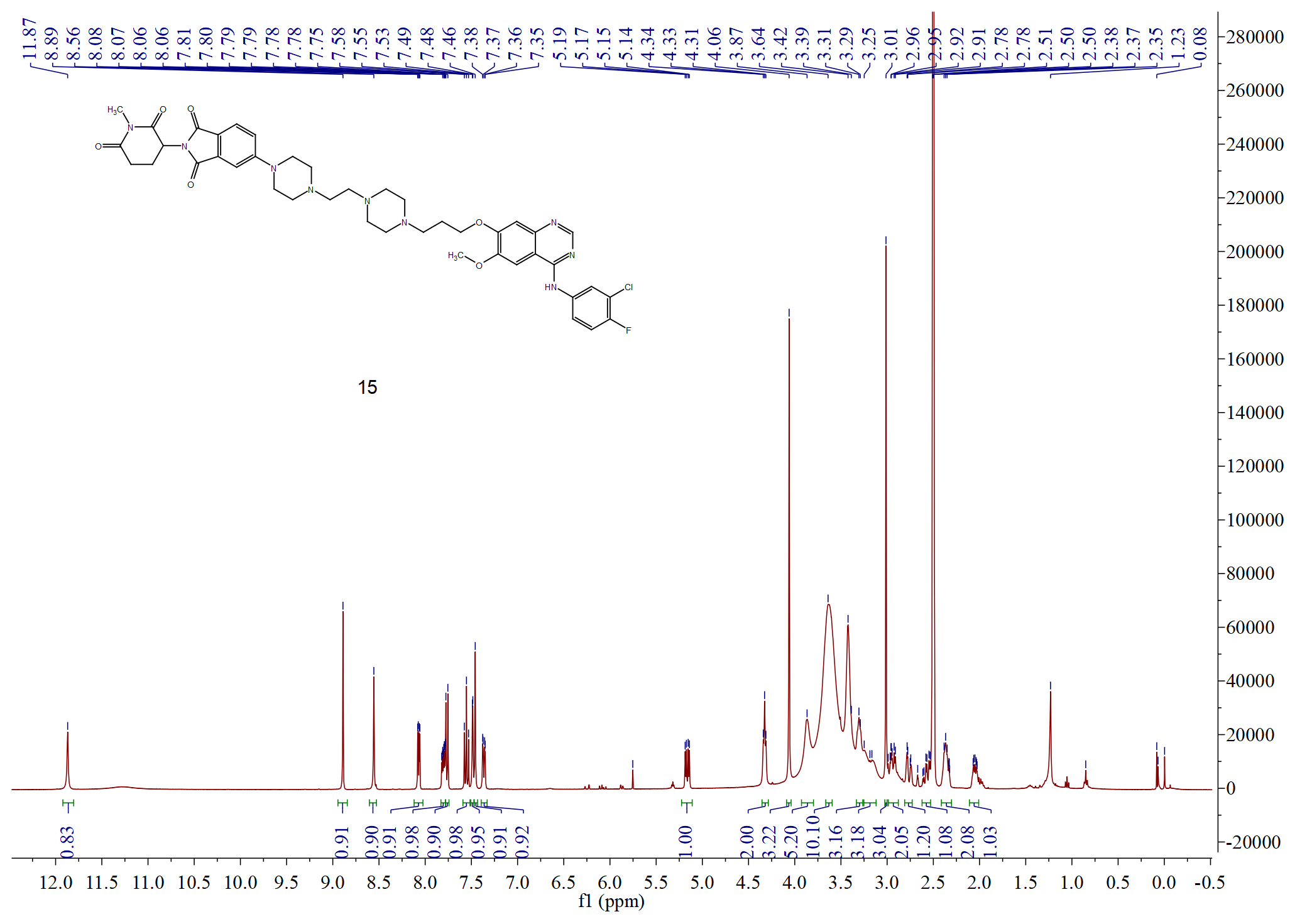


**7. HPLC traces of final compounds.**

HPLC compound 1, purity: 98.24%


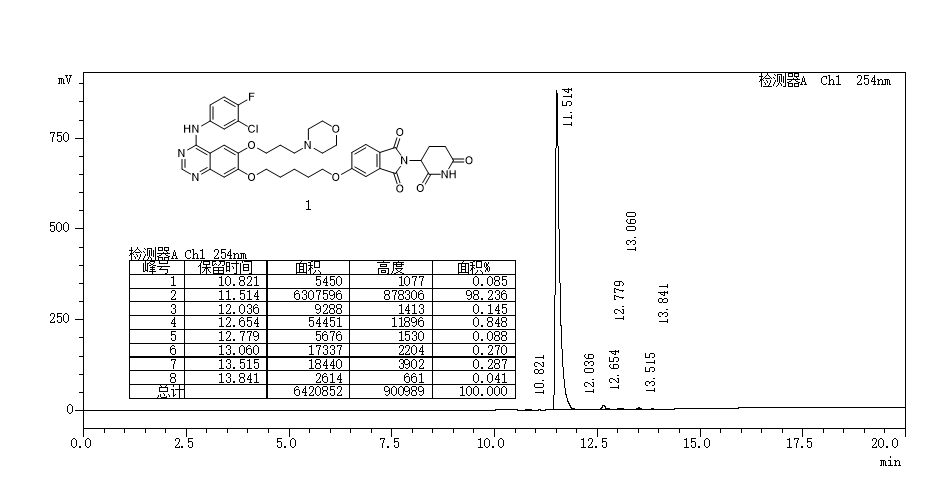


HPLC compound 2, purity: 96.39%


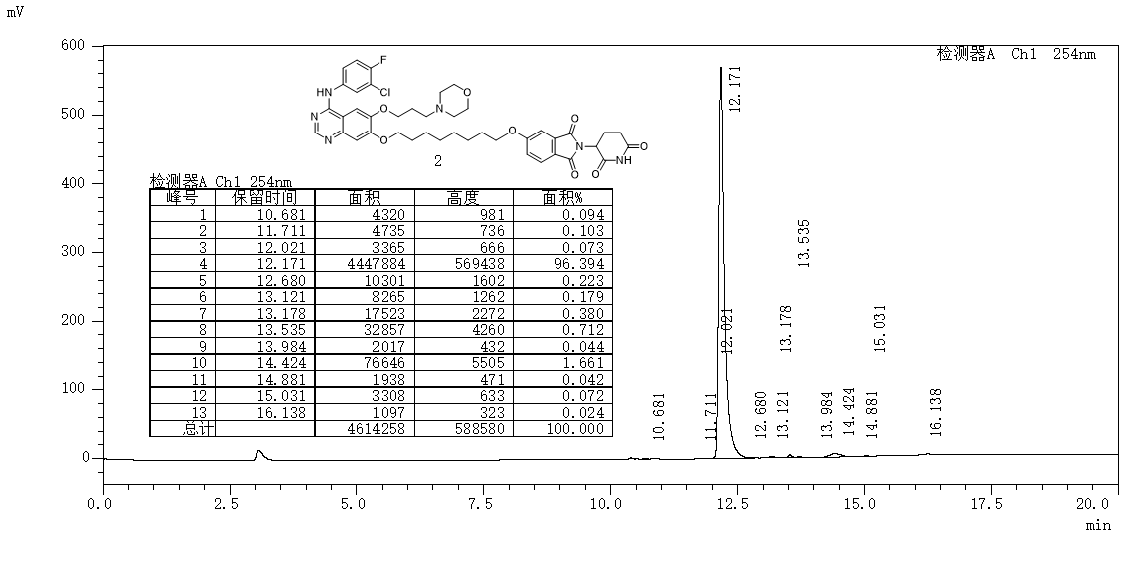


HPLC compound 3, purity: 98.47%


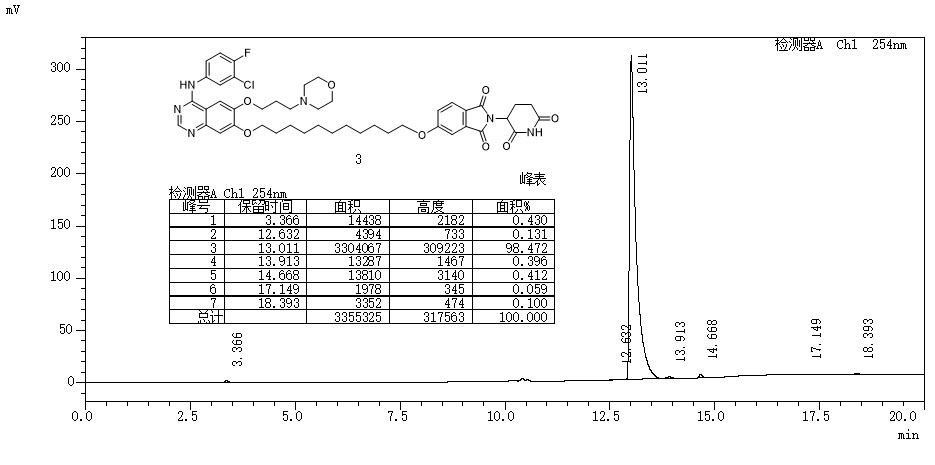


HPLC compound 4, purity: 95.06%


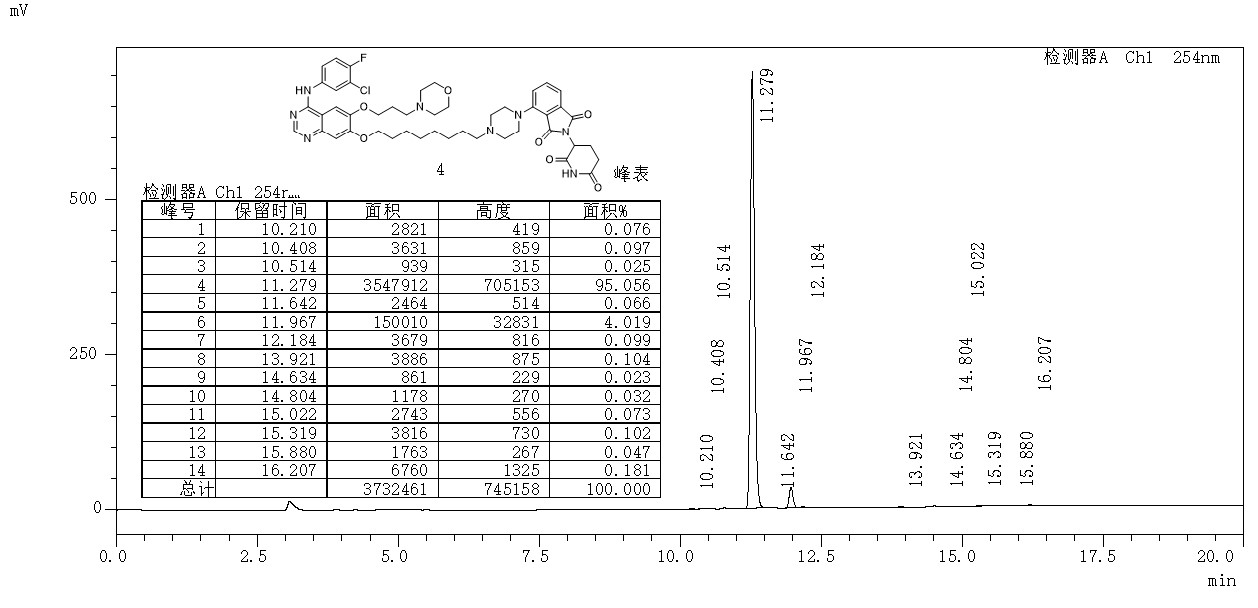


HPLC compound 5, purity: 97.59%


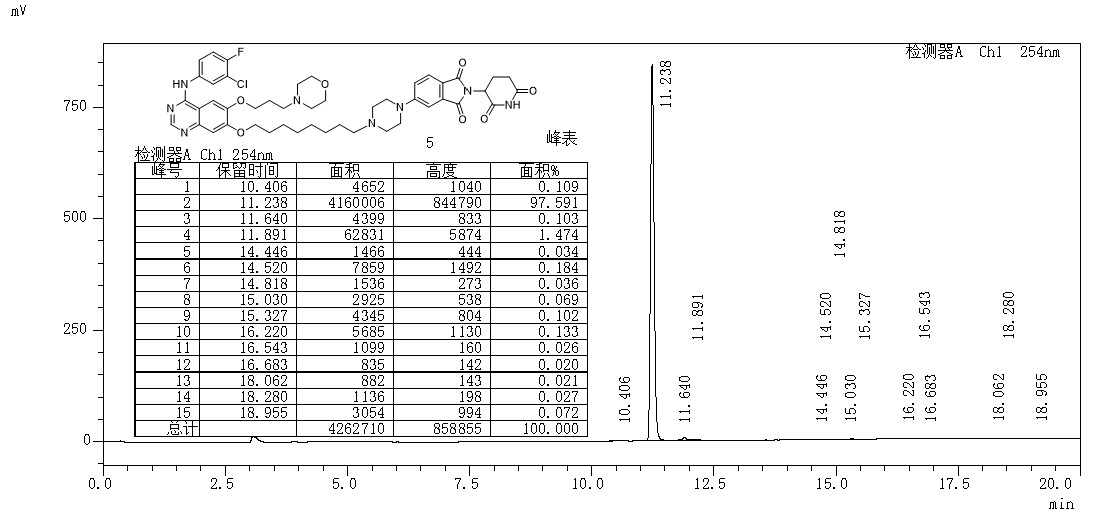


HPLC compound 6, purity: 97.75%


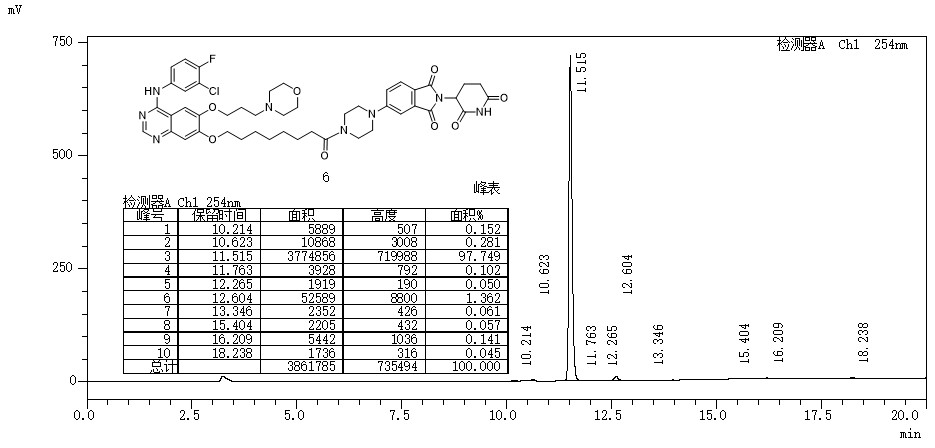


HPLC compound 7, purity: 95.12%


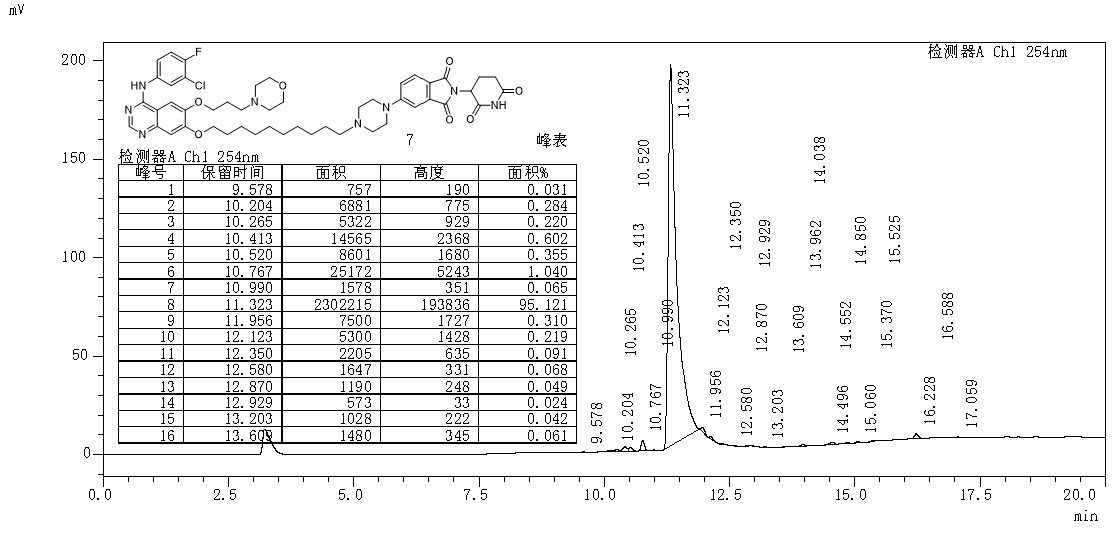


HPLC compound 8, purity: 99.93%


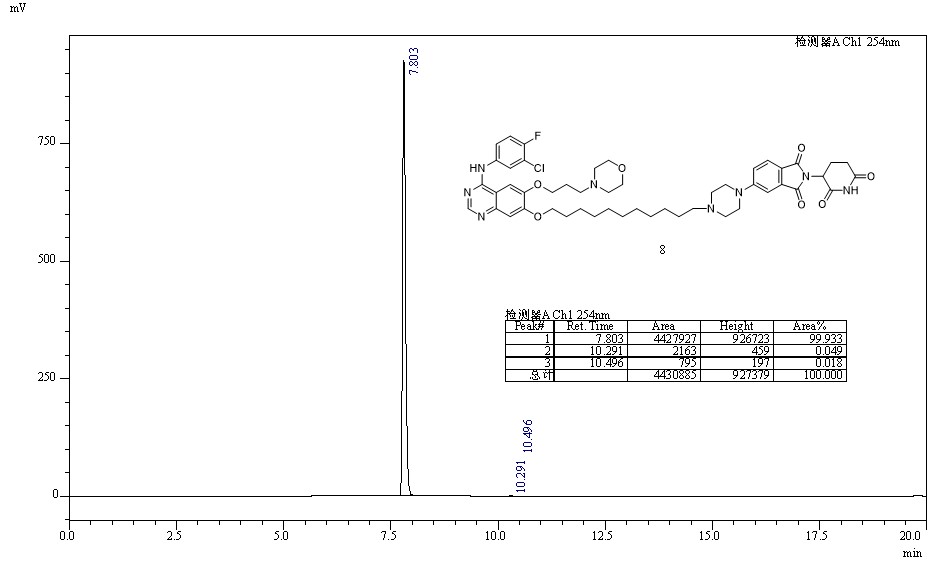


HPLC compound 9, purity: 96.23%


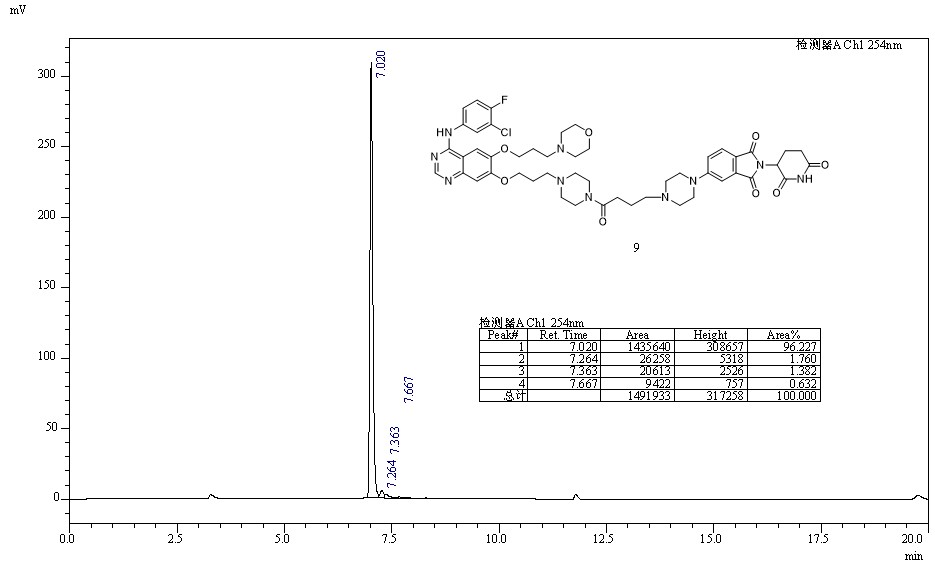


HPLC compound 10, purity: 99.96%


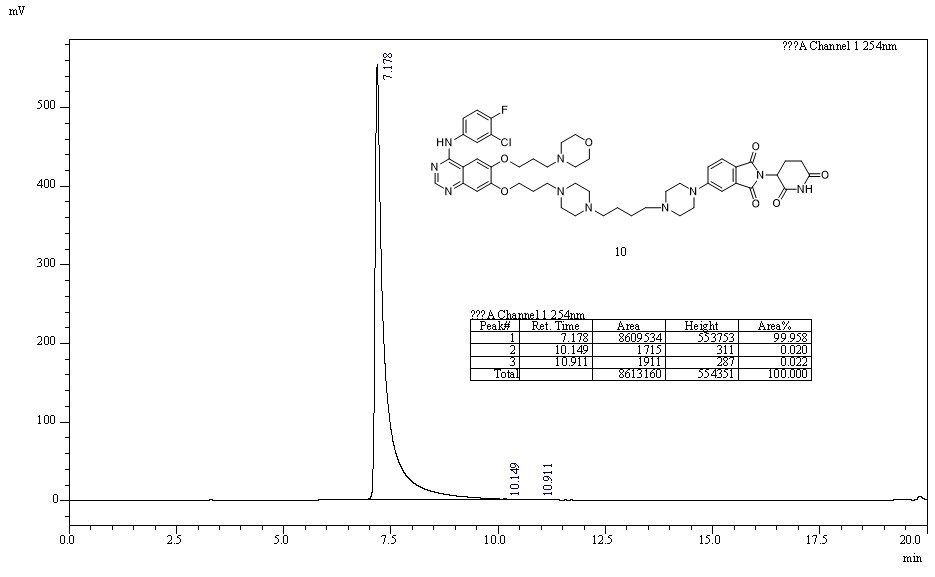


HPLC compound 11, purity: 99.45%


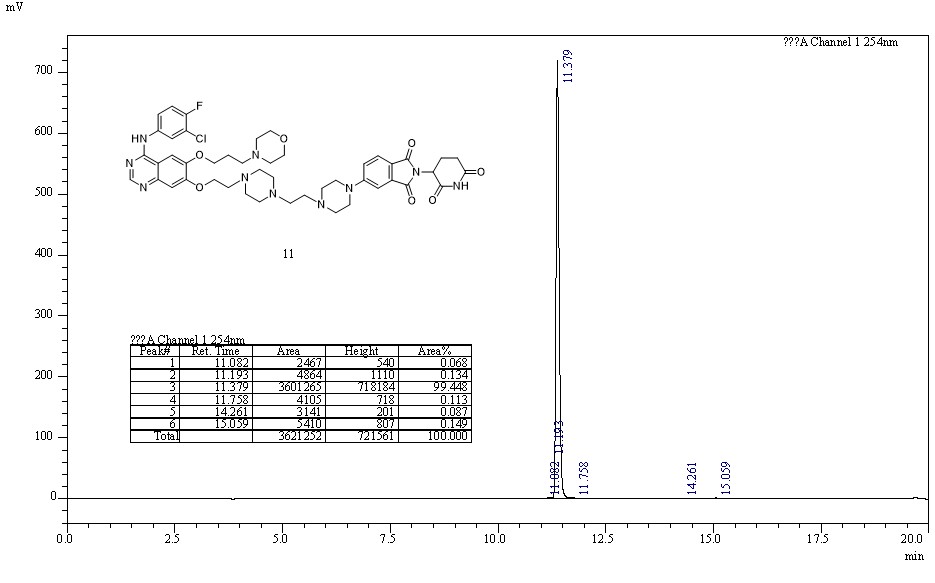


HPLC compound 12, purity: 98.85%


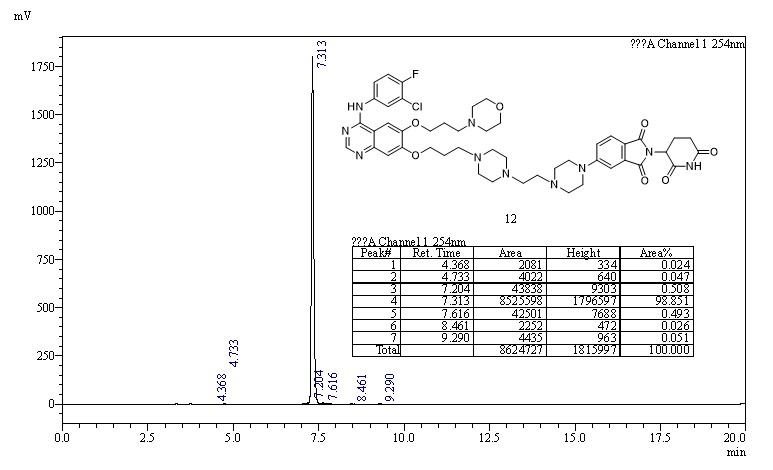


HPLC compound 13, purity: 98.15%


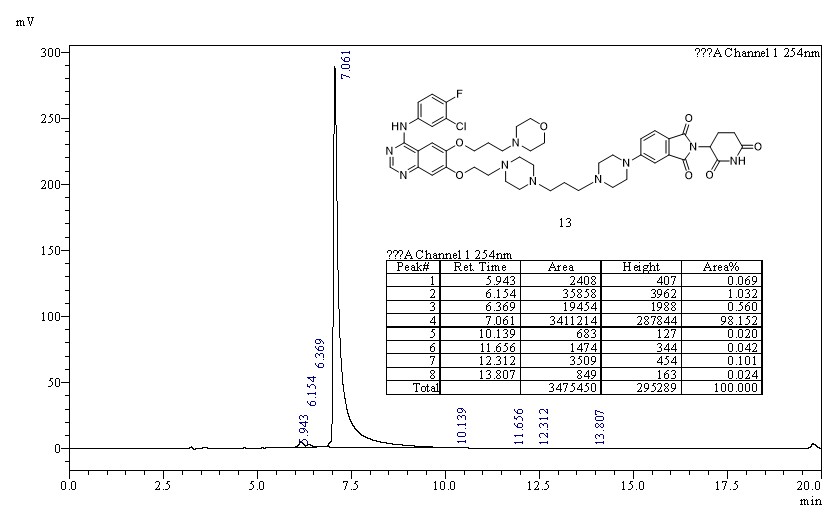


HPLC compound 14, purity: 99.51%


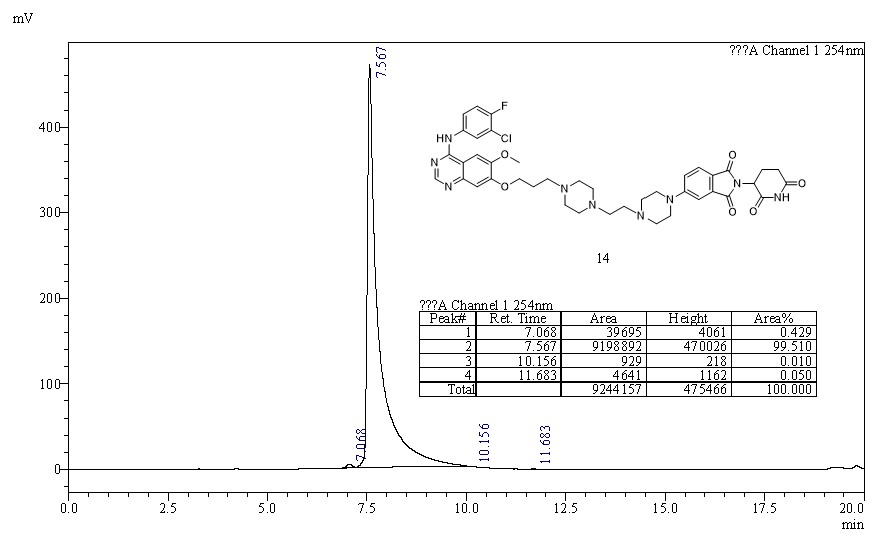


HPLC compound 15, purity: 99.42%


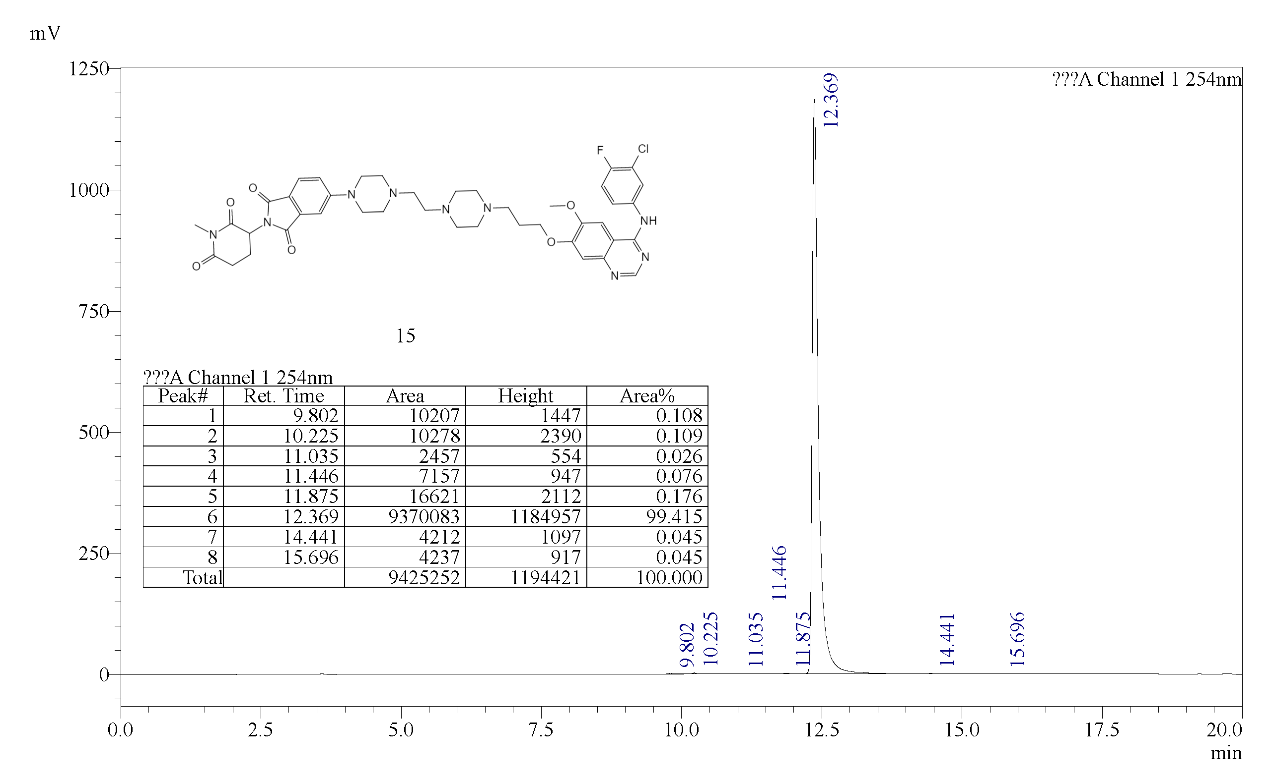


**8. NMR traces and LCMS data for MS154 purchased from MCE**


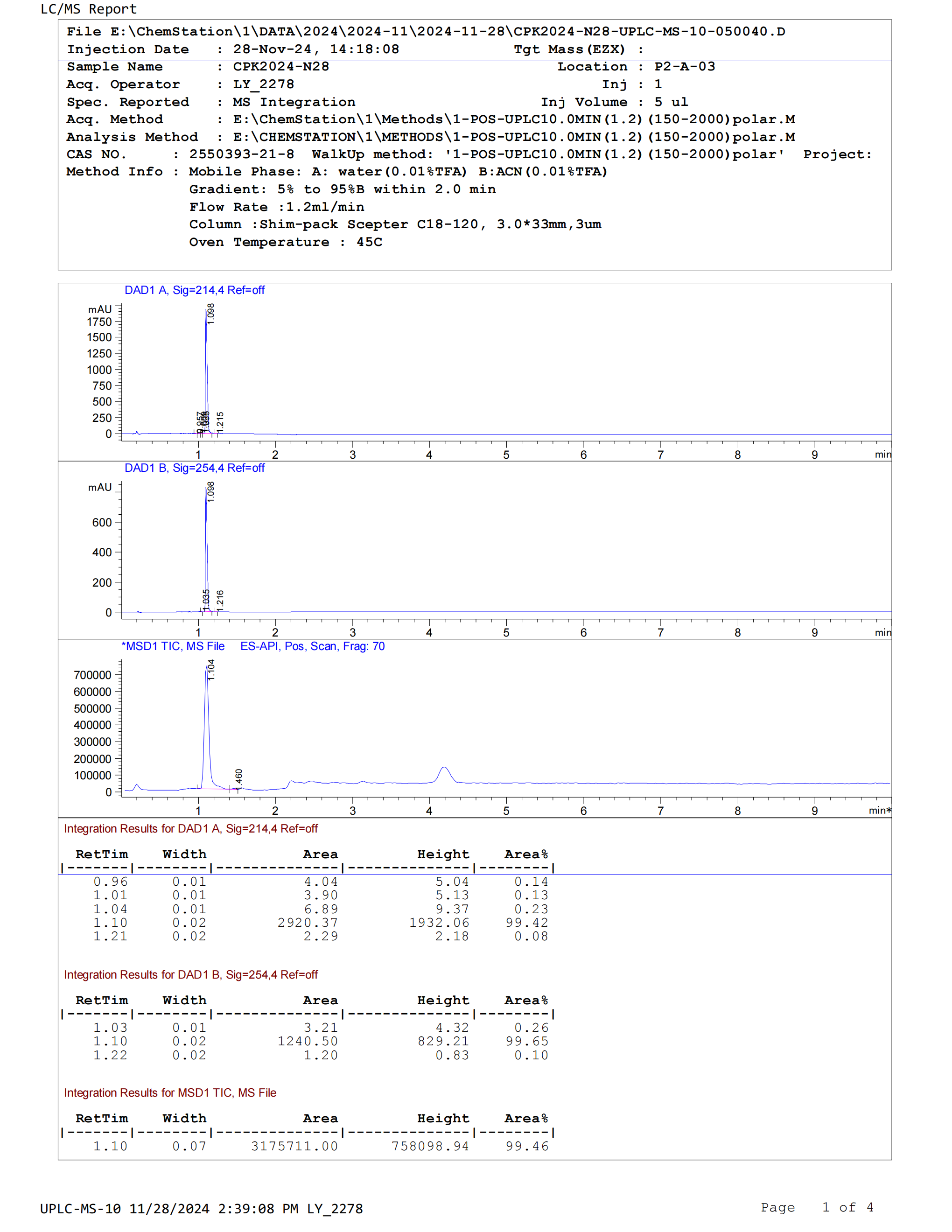


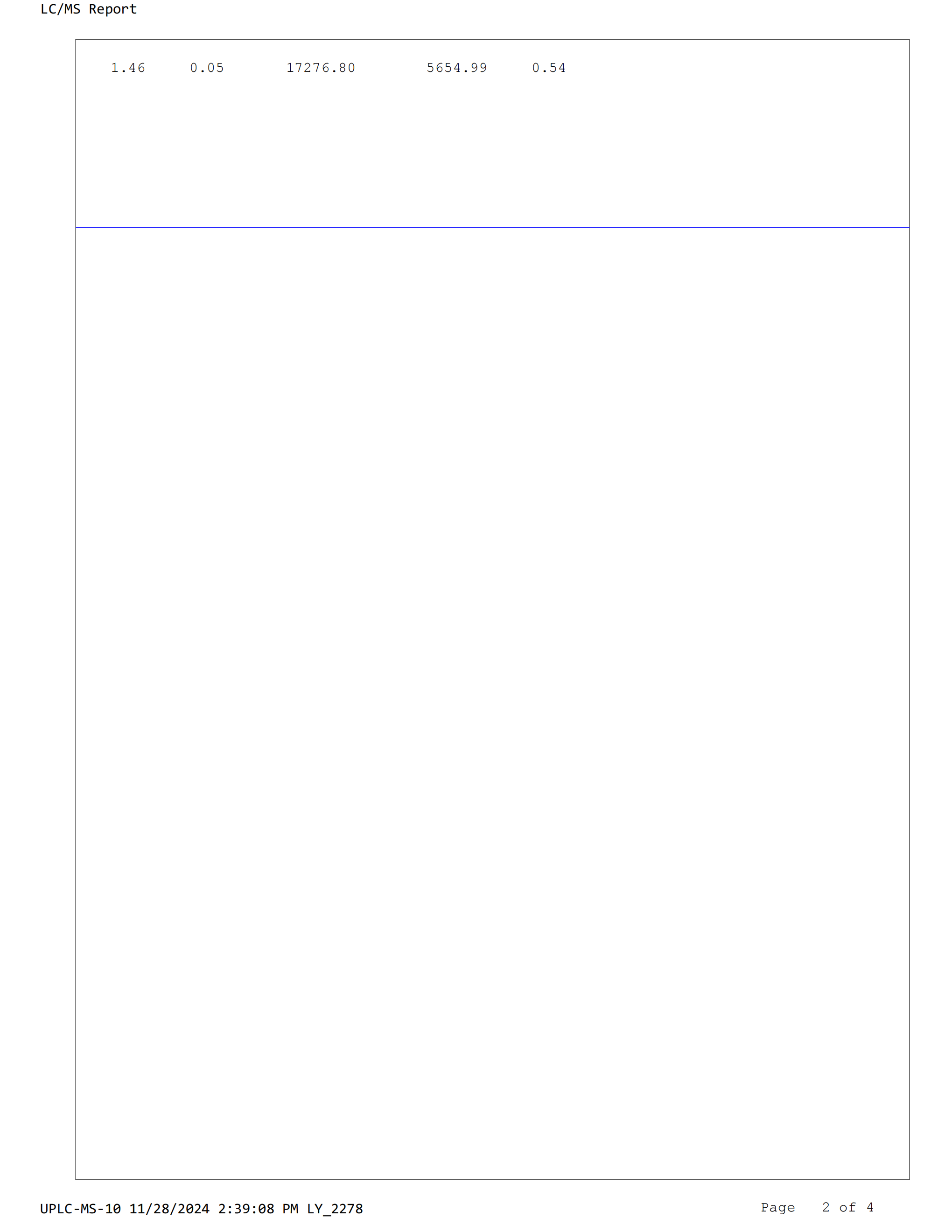


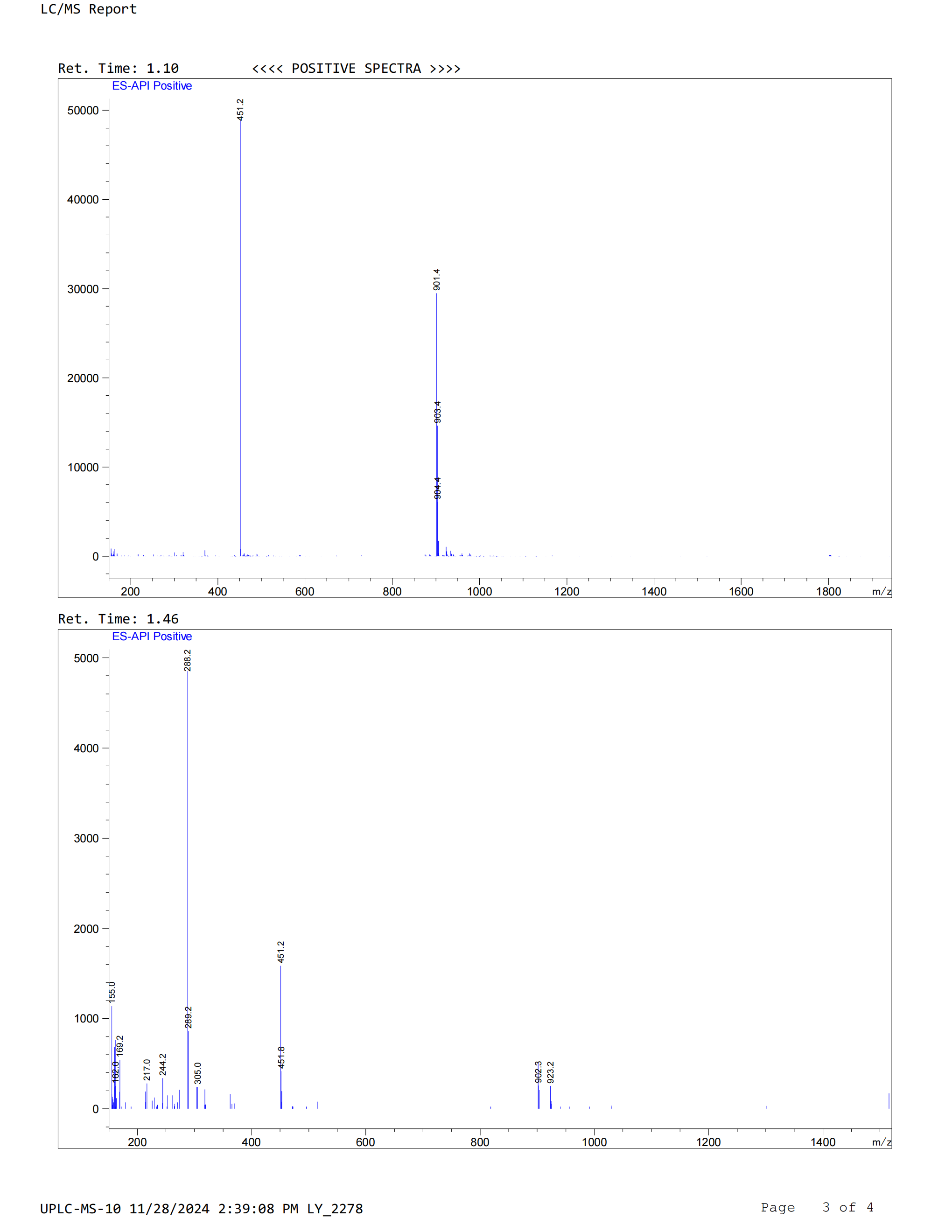

Supplement: Supplementary file 1 [file Supplementaryfile1.docx]
